# Clonal Hematopoiesis of Indeterminate Potential With Loss of *Tet2* Enhances Risk for Atrial Fibrillation Through *Nlrp3* Inflammasome Activation

**DOI:** 10.1161/CIRCULATIONAHA.123.065597

**Published:** 2024-02-15

**Authors:** Amy Erica Lin, Aneesh C. Bapat, Ling Xiao, Abhishek Niroula, Jiangchuan Ye, Waihay J. Wong, Mridul Agrawal, Christopher J. Farady, Andreas Boettcher, Christopher B. Hergott, Marie McConkey, Patricio Flores-Bringas, Veronica Shkolnik, Alexander G. Bick, David Milan, Pradeep Natarajan, Peter Libby, Patrick T. Ellinor, Benjamin L. Ebert

**Affiliations:** Division of Cardiovascular Medicine, Department of Medicine (A.E.L., P.L.), Brigham and Women’s Hospital, Boston, MA.; Department of Pathology (W.J.W., C.B.H.), Brigham and Women’s Hospital, Boston, MA.; Department of Medical Oncology, Dana Farber Cancer Institute, Harvard Medical School, Boston, MA (A.E.L., A.N., M.A., C.B.H., M.M.C., V.S., B.L.E.).; Cardiovascular Research Center (A.C.B., L.X., J.Y., D.M., P.N., P.T.E.), Massachusetts General Hospital, Boston.; Demoulas Cardiac Arrhythmia Service, Division of Cardiovascular Medicine, Department of Medicine (A.C.B., P.T.E.), Massachusetts General Hospital, Boston.; Broad Institute of Massachusetts Institute of Technology and Harvard, Cambridge (L.X., A.N., J.Y., P.F.-B., P.N., P.T.E., B.L.E.).; Department of Laboratory Medicine, Lund University, Sweden (A.N.).; Institute of Biomedicine, SciLifeLab, University of Gothenburg, Sweden (A.N.).; Novartis Institutes for BioMedical Research Forum 1, Basel, Switzerland (C.J.F., A.B.).; Division of Genetic Medicine, Department of Medicine, Vanderbilt University School of Medicine, Nashville, TN (A.G.B.).; Leducq Foundation, Boston, MA (D.M.).; Howard Hughes Medical Institute, Boston, MA (B.L.E.).

**Keywords:** atrial fibrillation, clonal hematopoiesis, inflammasome

## Abstract

**BACKGROUND:**

Clonal hematopoiesis of indeterminate potential (CHIP), a common age-associated phenomenon, associates with increased risk of both hematological malignancy and cardiovascular disease. Although CHIP is known to increase the risk of myocardial infarction and heart failure, the influence of CHIP in cardiac arrhythmias, such as atrial fibrillation (AF), is less explored.

**METHODS:**

CHIP prevalence was determined in the UK Biobank, and incident AF analysis was stratified by CHIP status and clone size using Cox proportional hazard models. Lethally irradiated mice were transplanted with hematopoietic-specific loss of *Tet2*, hematopoietic-specific loss of *Tet2* and *Nlrp3*, or wild-type control and fed a Western diet, compounded with or without NLRP3 (NLR [NACHT, LRR {leucine rich repeat}] family pyrin domain containing protein 3) inhibitor, NP3-361, for 6 to 9 weeks. Mice underwent in vivo invasive electrophysiology studies and ex vivo optical mapping. Cardiomyocytes from *Ldlr*^−/−^ mice with hematopoietic-specific loss of *Tet2* or wild-type control and fed a Western diet were isolated to evaluate calcium signaling dynamics and analysis. Cocultures of pluripotent stem cell–derived atrial cardiomyocytes were incubated with *Tet2*-deficient bone marrow–derived macrophages, wild-type control, or cytokines IL-1β (interleukin 1β) or IL-6 (interleukin 6).

**RESULTS:**

Analysis of the UK Biobank showed individuals with CHIP, in particular *TET2* CHIP, have increased incident AF. Hematopoietic-specific inactivation of *Tet2* increases AF propensity in atherogenic and nonatherogenic mouse models and is associated with increased Nlrp3 expression and CaMKII (Ca2+/calmodulin-dependent protein kinase II) activation, with AF susceptibility prevented by inactivation of *Nlrp3*. Cardiomyocytes isolated from *Ldlr*^−/−^ mice with hematopoietic inactivation of *Tet2* and fed a Western diet have impaired calcium release from the sarcoplasmic reticulum into the cytosol, contributing to atrial arrhythmogenesis. Abnormal sarcoplasmic reticulum calcium release was recapitulated in cocultures of cardiomyocytes with the addition of *Tet2*-deficient macrophages or cytokines IL-1β or IL-6.

**CONCLUSIONS:**

We identified a modest association between CHIP, particularly *TET2* CHIP, and incident AF in the UK Biobank population. In a mouse model of AF resulting from hematopoietic-specific inactivation of *Tet2*, we propose altered calcium handling as an arrhythmogenic mechanism, dependent on *Nlrp3* inflammasome activation. Our data are in keeping with previous studies of CHIP in cardiovascular disease, and further studies into the therapeutic potential of NLRP3 inhibition for individuals with *TET2* CHIP may be warranted.

**Clinical Perspective**

**What Is New?**

- Existing literature links clonal hematopoiesis of indeterminate potential (CHIP) with atherosclerotic heart disease. Here, we demonstrate that individuals with CHIP, specifically those with large variant allele frequency tet methylcystosine dioxygenase 2 (*TET2*) mutations, have an increased risk for atrial fibrillation (AF).
- Hematopoietic-specific *Tet2* deficiency leads to increased AF susceptibility in mice mediated by increased Nlrp3 (NLR [NACHT, LRR {leucine rich repeat}] family pyrin domain containing protein 3) inflammasome expression, which leads to increased CaMKII (Ca2^+^/calmodulin-dependent protein kinase II) activation and cardiomyocyte calcium dysregulation.
- Targeting Nlrp3 inflammasome activation prevents *Tet2* deficiency–related arrhythmogenesis in a mouse model.

**What Are the Clinical Implications?**

- These data add to our growing understanding of AF pathogenesis, particularly the role of immune cells and the inflammasome.
- Individuals with *TET2*-mutated CHIP may carry an elevated risk of AF compared with the general population, making CHIP a potential novel biomarker for increased risk of AF.
- CHIP mutation status can allow for the identification of a patient population that may be responsive to the treatment of AF with an NLRP3 inhibitor.

Clonal hematopoiesis of indeterminate potential (CHIP), an age-associated phenomenon, results from somatic leukemogenic driver mutations in hematopoietic stem cells that generate clonal populations in the peripheral blood.^1,2^ CHIP is common, occurring in >10% of individuals older than 70 years of age, and increases the risk of myeloid malignancies. In addition, CHIP has emerged as an unexpected and important risk factor for cardiovascular disease (CVD).^1–3^ Individuals with CHIP have an increased risk of myocardial infarction and ischemic stroke, independent of traditional CVD risk factors.^3^ Atherosclerosis-prone *Ldlr*^−/−^ mice with genetic inactivation in myeloid cells of tet methylcystosine dioxygenase 2 (*Tet2*), a gene commonly mutated in CHIP, develop accelerated atherosclerosis with increased proinflammatory cytokine production including IL-1β (interleukin 1β) and IL-6 (interleukin 6).^3,4^ The NLRP3 (NLR [NACHT {NAIP (neuronal apoptosis inhibitor protein), C2TA (class 2 transcription activator of the MHC), HET-E (heterokaryon incompatibility), and TP1 (telomerase-associated protein)}, LRR {leucine rich repeat}] family pyrin domain containing protein 3) inhibitor MCC950 has been reported to reverse these effects, pointing to aberrant inflammation as a contributor to the mechanism of aggravated atherogenesis.^4^ Additional studies continue to broaden our understanding of the role of CHIP in CVD and show that the presence of CHIP leads to accelerated and worsened outcomes in heart failure (HF) and severe aortic stenosis after transcatheter aortic valve implantation.^5,6^ However, the role of CHIP in arrhythmias, specifically atrial fibrillation (AF), is less understood.

Worldwide, AF is the most prevalent sustained arrhythmia and causes substantial morbidity and mortality.^7^ AF pathophysiology is multifactorial, and many risk factors have been identified including advancing age, atherosclerotic heart disease, valvulopathies, atrial and ventricular remodeling, hypertension, and diabetes. In addition, inflammation contributes to AF and associated increased prothrombotic risk.^8^ Patients with AF often have elevated circulating proinflammatory cytokines IL- β, IL-6, and TNFα (tumor necrosis factor α) and increased activation of the NLRP3 inflammasome.^8,9^ Mice with constitutively active *Nlrp3*^A350V^ overexpressed in cardiomyocytes recapitulate AF inducibility and susceptibility, thus establishing a role for aberrant cardiac inflammasome responses in AF.^9^ We hypothesized that patients with CHIP have an increased risk for AF, resulting in part from increased inflammation, mediated by aberrant and overactive NLRP3 inflammasome activation in cardiac macrophages.

## METHODS

### Data Availability

Additional and detailed methods are included in the Supplemental Material. Genetic sequencing data analyzed are available through the UK Biobank Data Showcase. Data supporting the findings of this study are available from the corresponding authors upon reasonable request.

### Study Samples

In this study, we included 453 515 individuals with available whole-exome sequencing data in the UK Biobank from the existing >500 000 participants 40 to 70 years of age recruited between 2006 and 2010.^10,11^ After excluding individuals with prevalent hematological malignancies, missing covariates, and one of the third-degree relatives,^10,12^ 362 440 individuals remained in the cohort. Further, we excluded 4343 individuals with prevalent AF, leaving 358 097 individuals in the final analysis cohort. Using the whole-exome sequencing data, we identified CHIP, defined as the presence of somatic mutations in hematopoietic cells with a variant allele frequency (VAF) of ≥2%, as previously described.^13,14^ Incident AF analysis stratified by CHIP status and clone size (larger clone size defined as ≥10% VAF) was performed using Cox proportional hazard models adjusting for age, age^2^, sex (male and female), ever-smoked status (yes or no), genetic ancestry (European/non-European), body mass index (<30/≥30), prevalent type 2 diabetes, prevalent hypertension, prevalent coronary artery disease (CAD), and LDL (low-density lipoprotein; adjusted for statin use), as previously described.^15^ Individuals with AF, type 2 diabetes, hypertension, CAD, HF, valvular disease, and hematological malignancies were identified on the basis of the *International Classification of Diseases, Ninth* and *10th Revisions* (ICD-9, ICD-10), and Office of Population Censuses and Surveys Classification of Interventions and Procedures-4 (OPCS-4) codes (Table S1). The samples were followed up for up to 12 years from the time of recruitment to the UK Biobank through March 2020. The individuals diagnosed with AF ICD-9, ICD-10 codes or OPCS-4 for AF ablation were considered events, and individuals who did not experience events during the follow-up period were censored at the end of follow-up. Individuals who died before the end of the follow-up period were censored at death. For the secondary analysis, individuals with incident HF before AF diagnosis were censored at the time of HF diagnosis.

### Mouse Models

Experimental mouse strains include *Tet2*-floxed *B6;129S-Tet2^tm1.1laai/J^* (Jax, 017573), DNA (cytosine-5)-methyltransferase 3A (*Dnmt3a*)-floxed line *B6;129S4-Dnmt3a^tm3.1Enl/J^*,^16^ *Nlrp3*^−/−^ B6N.129-*Nlrp3^tm1Hhf/J^* (Jax, 017971), CD45.1 *B6.SJL-Ptprc*^a^ *Pepc*^b^*/BoyJ* (Jax, 002014), *Ldlr*^−/−^ B6129S7-*Ldlr^tm1Her/J^* (Jax, 002207), and BL6 C57BL6/J (Jax, 000664). To generate the *Tet2* knockout specific or *Dnmt3a* knockout specific to the entire hematopoietic lineage, *Vav1-Cre^+^* recombinase mice (B6.Cg-*Commd10*^*Tg(Vav1-icre)A2Kio/J*^ [Jax, 008610]) were crossed with the floxed line. Wild-type (WT) *Vav1-Cre^+^* animals were used as controls. To generate *Tet2* hematopoietic-specific and *Nlrp3* double knockout, *Vav1-Cre^+^* mice were crossed. To generate transplant recipients, *Ldlr*^−/−^ and *Nlrp3*^−/−^ lines were backcrossed to CD45.1 mice.^3^ All alleles were genotyped by Transnetyx, Inc. All animals were maintained according to the approved grant by institutional animal care and use committee of Brigham and Women’s Hospital and Dana Farber Cancer Institute. Animals were housed with a standard light-dark 12 hour:12 hour schedule, with ad libitum access to food and water. Mice were maintained on normal chow diet, and after confirmation of engraftment at 4 weeks after transplant, mice were fed a Western diet (WD) consisting of 21% milk fat, 1.2% cholesterol (WD; Envigo TD.96121 or Research Diets D12018C) plus NLRP3 inhibitor (NP3-361 180 mg/kg chow; Novartis) for a minimum of 5 weeks.

### In Vivo Electrophysiology Studies

Electrophysiology studies were performed using established protocols.^17–19^ An octapolar catheter (EPR-800, Millar) was positioned in the right atrium and ventricle, with position ascertained by continuous monitoring of the intracardiac electrograms. Sinus node function was determined by measuring the sinus node recovery time after 30 s of pacing at 2 cycle lengths (100 and 80 ms). Wenckebach cycle length was determined with progressively faster atrial pacing rates. Atrial, ventricular, and atrioventricular nodal refractory periods were measured using programmed electrical stimulation with overdrive pacing trains at 100 ms followed by single extra stimuli. Provocative testing for arrhythmia induction was performed with rapid burst pacing at gradually faster rates (starting at 50 ms) to a pacing cycle length of 20 ms and lasting 3 and 6 s. AF was defined as a rapid atrial rhythm with atrial rate greater than ventricular rate and irregular ventricular response (R-R intervals). Each mouse received 36 provocative stimuli with burst pacing. The duration of AF was measured from the end of the pacing train to the end of the rapid atrial activity. Mice were considered to have AF if they had >1 episode of AF for >250 ms in duration. To measure percentage of AF inducibility, a higher threshold of 1 s was used, and the total number of AF episodes lasting >1 s for each mouse was divided by the total number of provocative maneuvers.

### Optical Mapping

Isolation and perfusion of the heart were performed as previously described.^17,20^ Excised and cannulated hearts were perfused with a modified Tyrode solution containing 1.3 mM CaCl_2_, using a Langendorff perfusion setup. Blebbistatin (10 mM, Tocris Bioscience) was used to arrest cardiac motion. Hearts were stained for 30 minutes with a voltage-sensitive dye (di-4-ANEPPs, 2 mmol/L in DMSO, Invitrogen). Custom-made epicardial platinum electrodes and a Medtronic stimulator were used to pace the heart at 60- to 120-ms cycle lengths at twice the capture threshold (4-ms square wave stimuli). A halogen light source (X-Cite 150 W, filtered at 520±45 nm) was used to excite fluorescence. Emissions >610 nm were collected and focused onto an 80×80 charge-coupled device camera (RedShirt Imaging SMQ Camera, Macroscope IIA) using a 50-mm original magnification ×2.7 lens (numerical aperture 0.4). Data sampling was performed at 2000 frames/s with a filter setting of 1 kHz. A specifically designed MATLAB program was used to perform data analysis to generate conduction velocities and action potential duration (APD) at 50%, 70%, and 90% repolarization.

### Calcium Imaging

Isolated atrial cardiomyocytes were loaded with calcium indicator Fluo-3 AM (4.4 µmol/L, Invitrogen F1241) for 10 minutes followed by a 30-minute washout, or Fluo-4 AM non-wash dye (2.5 μmol/L, Invitrogen F36206) for 30 minutes in Tyrode’s solution in the presence of 0.02% Pluronic F-127 at room temperature, before imaging. Pacing at 1 Hz was used, and 10 mmol/L caffeine was applied to assess sarcoplasmic reticulum (SR) calcium load. Spontaneous calcium release frequency in diastole was calculated using Fluo-3 AM fluorescence, obtained with a fluorescein isothiocyanate filter set (excitation, HQ480; mirror, Q505LP; emission, HQ535/50 m; Chroma) and an X-Cite Exacte mercury arc lamp (Luman Dynamics). Fluorescent images were recorded with a Nikon Eclipse Ti-U inverted microscope (Nikon Instruments Inc), a NeuroCCDSM camera, and Neuroplex software (RedShirt Imaging). Calcium transients were analyzed with Neuroplex and Clampfit 9.2 (Molecular Devices Inc). High-throughput optical fluorescent time series images of Fluo-4 NW fluorescence were acquired at 100 Hz using the IC200 KIC instrument and image analysis with the CyteSeer (Vala Sciences) as previously described.^21^

### Data and Statistical Analyses

GraphPad Prism software v9.3.1 was used for statistical analyses. Statistical significance was assessed by the Student *t* test (unpaired, 2-tailed) for 2-group, normally distributed data. AF inducibility was assessed by the Fisher exact test or χ^2^ test if there were 3 groups. Linear regression was used to assess APD dependence on clonal expansion. Calcium studies of isolated murine cardiomyocytes used linear mixed models, with genotype set as fixed experiment effects and mouse genotype blocked to account for random effects. For multigroup analysis, 1-way ANOVA with Tukey multiple comparisons was used. UK Biobank cohort data were analyzed using Cox proportion hazard models adjusted for age, age^2^, sex, smoking history, genetic ancestry, body mass index, type 2 diabetes, hypertension, CAD, and LDL, as previously described.^15^ *P* values ≤0.05 were considered significant. All figure plots show mean with error bars reflecting SEM, unless otherwise stated.

## RESULTS

### CHIP Is Associated With Risk of AF

**Figure 1.**
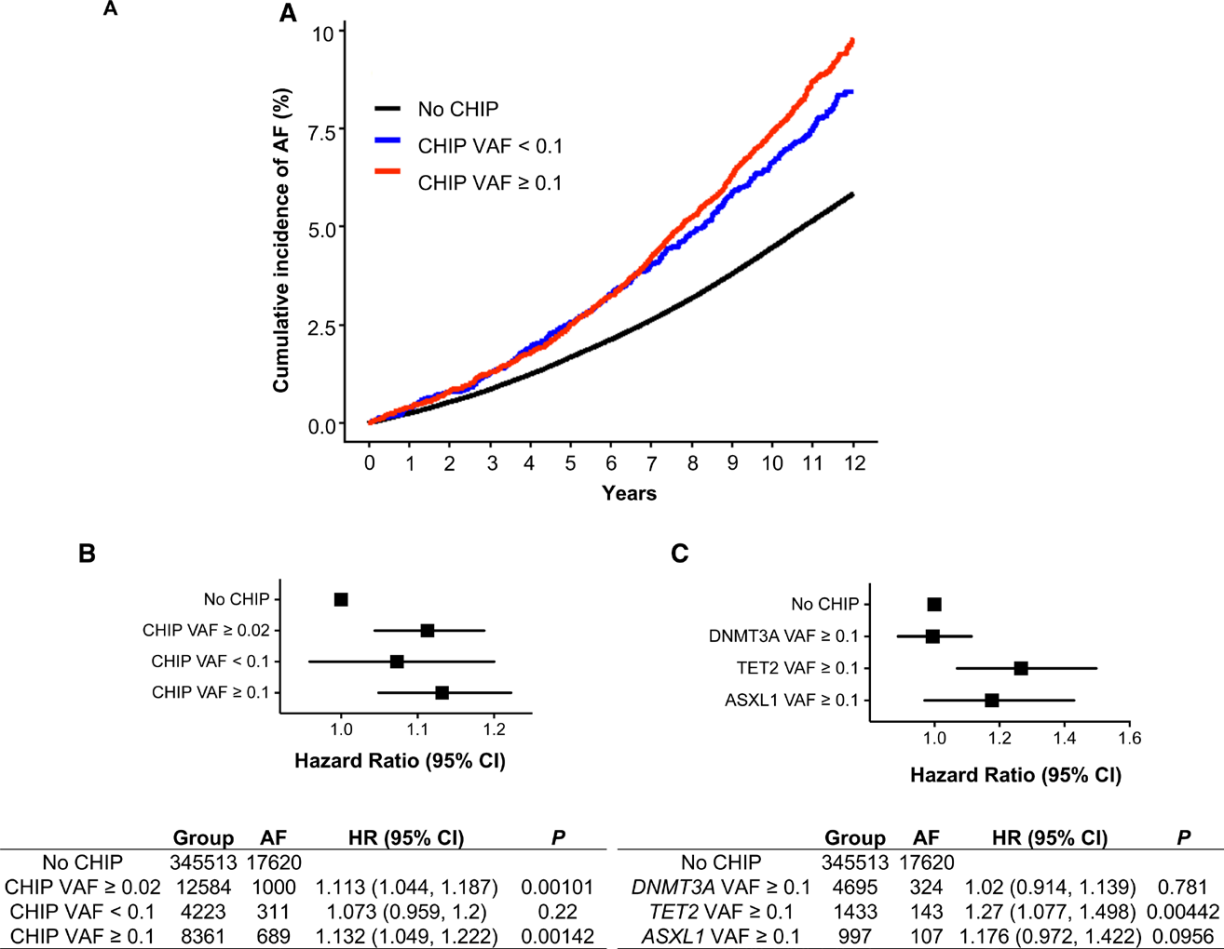
***TET2* CHIP is associated with increased risk of incident AF in UK Biobank.** A total of 358 097 individuals from the UK Biobank with available whole-exome sequencing were identified. Prevalent hematological malignancies, valvular heart disease, HF, and prevalent AF were excluded. Cox proportion hazard models adjusted for age, age^2^, sex, ever-smoked status, genetic ancestry (European/non-European), BMI, prevalent type 2 diabetes (T2D), prevalent hypertension, prevalent coronary artery disease (CAD), LDL (adjusted for statin use), as previously described.^15^ Individuals with AF, T2D, hypertension, CAD, HF, valvular disease, and hematological malignancies were identified on the basis of *International Classification of Diseases, Ninth* and *10th Revisions*, codes. **A** and **B**, Increased cumulative incidence of AF in individuals with CHIP ≥0.1, with HR 1.132 (95% CI, 1.049–1.222; *P*=0.00142). **C**, Increased cumulative incidence of AF in patients with TET2 CHIP ≥0.1 with HR 1.27 (95% CI, 1.077–1.498; *P*=0.00442). AF indicates atrial fibrillation; ASXL1, additional sex combs-like 1; BMI, body mass index; CHIP, clonal hematopoiesis of indeterminate potential; DNMT3A, DNA (cytosine-5)-methyltransferase 3A; HF, heart failure; HR, hazard ratio; LDL, low-density lipoprotein; TET2, tet methylcystosine dioxygenase 2; and VAF, variant allele frequency.

To determine the relationship between CHIP and AF, we analyzed whole-exome sequencing data and health data from the UK Biobank (n=358 097), a prospective cohort with genetic, demographic, and health-related data. Individuals with prevalent AF, valvular heart disease (mitral stenosis, mitral valve repair, replacement, or valvotomy), HF, or prevalent hematological malignancy before DNA collection were excluded (Table S1). In total, CHIP with VAF ≥0.02 was identified in 12 584 individuals, and the most commonly mutated CHIP driver genes were *DNMT3A*, *TET2*, and additional sex combs-like 1 (*ASXL1*; Figure S1).^1–3,5,15,22–24^ In Cox proportion hazard models adjusted for age, age^2^, sex, smoking history, genetic ancestry, body mass index, type 2 diabetes, hypertension, CAD, and LDL,^15^ the presence of CHIP significantly associated with incident AF (hazard ratio [HR], 1.113 [95% CI, 1.044–1.187]; *P*=0.001; Figure 1A and 1B). Individuals with expanded CHIP clones (VAF ≥0.1) have a greater risk of developing malignant and nonmalignant manifestations, including CVD.^1–3,25^ In this analysis, individuals with expanded CHIP clones had a higher incidence of AF compared with individuals without CHIP (HR, 1.132 [95% CI, 1.049–1.222]; *P*=0.00142; Figure 1A and 1B). In comparison, smaller CHIP clones (VAF <0.1) did not associate significantly with incident AF (HR, 1.07 [95% CI, 0.96–1.2]; *P*=0.22; Figure 1A and 1B). Gene-specific analysis showed variable risk of AF. Although *TET2* mutations with a VAF ≥0.1 showed the strongest association with incident AF compared with those without CHIP (HR, 1.27 [95% CI, 1.077–1.498]; *P*=0.0044), *DNMT3A* or *ASXL1* mutations at the same VAF did not associate significantly (HR, 1.02 [95% CI, 0.914–1.139]; *P*=0.718; HR, 1.176 [95% CI, 0.972–1.422]; *P*=0.096, respectively; Figure 1C).

Previous studies have associated HF with AF as well as CHIP.^5,26,27^ To investigate whether the relationship between CHIP and AF depends on HF, we performed a secondary analysis restricted to individuals with incident HF diagnosis censored at the time of HF diagnosis. We found that the expanded CHIP clones and expanded large *TET2* clones were associated with increased incident AF (CHIP: HR, 1.108 [95% CI, 1.038–1.182]; *P*=0.00213; CHIP VAF ≥0.1: HR, 1.119 [95% CI, 1.034–1.209]; *P*=0.005; *TET2*: HR, 1.266 [95% CI, 1.07–1.497]; *P*=0.00591) compared with *DNMT3A* or *ASXL1* mutations, which showed no significant associations (HR, 0.995 [95% CI, 0.888–1.114]; *P*=0.931; and HR, 1.177 [95% CI, 0.97–1.429]; *P*=0.099, respectively; Figure S2A through S2C). These results indicate that individuals with CHIP driven by larger VAF clones, and particularly those with *TET2* mutations, have an increased risk of AF independent of HF and further support the finding that AF risk varies among specific CHIP mutations.

### Hematopoietic-Specific Loss of *Tet2* Promotes AF in Atherosclerosis-Prone Mice

**Figure 2.**
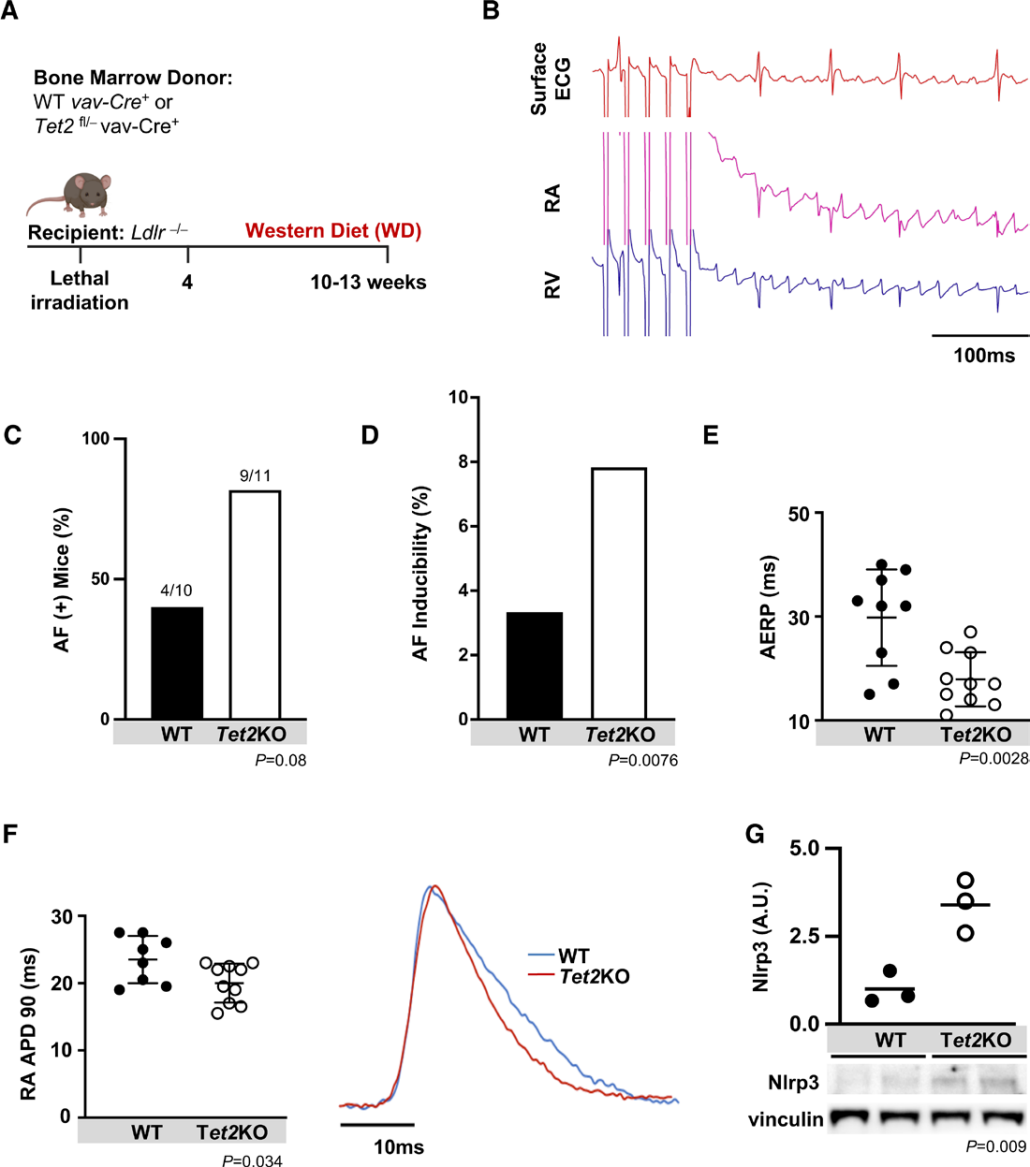
**Hematopoietic-specific inactivation of *Tet2* augments AF inducibility in *Ldlr^−/−^ mice*. A**, Lethally irradiated *Ldlr*^−/−^ mice transplanted with bone marrow with hematopoietic-specific inactivation of *Tet2* (*Tet2*KO) or WT controls and fed Western diet (WD) for 6 to 9 weeks. **B**, Representative tracing of pacing-induced AF, showing surface ECG, right atrial (RA) and right ventricle (RV) intracardiac electrograms. **C**, Hematopoieticspecific inactivation of *Tet2* resulted in an increased number of mice inducible for AF (number of mice with AF/total mice in the group). **D**, Increased AF inducibility was seen in *Tet2*KO mice. Percentage reflects ratio of total number of provoked AF episodes to total programmed electrical provocations. **E**, Atrial effective refractory period (AERP) measured at a drivetrain of 100 ms, demonstrating shortening in *Tet2*KO recipients. **F**, Optically derived right atrial action potential duration at 90% repolarization (RA APD90). Representative optical action potential tracing to right. **G**, Relative atrial protein expression of Nlrp3, with representative blots below, showing >3-fold increase in Nlrp3 expression. Western blot quantification relative to vinculin loading control and then normalized. Statistical testing: Fisher exact test for **C** and **D**; 2-tailed Student *t* test for **E** through **G**. Mean (SD) shown for **E** and **F**. AF indicates atrial fibrillation; A.U., arbitrary units; KO, knockout; LDLR, low-density lipoprotein receptor; Nlrp3, NLR (NACHT, LRR [leucine rich repeat]) family pyrin domain containing protein 3; TET2, tet methylcystosine dioxygenase 2; and WT, wild-type.

To assess the causality of the impact of CHIP on AF in carefully controlled murine experiments, we generated mice with hematopoietic-specific loss of the common CHIP gene, *Tet2*. Because mice do not typically develop spontaneous AF, elicitation requires electrical stimulation and alterations such as obesity or atherosclerosis-promoting diets.^17,20,28–30^ As an initial step, we examined the effect of *Tet2* inactivation in hematopoietic cells in atherosclerosis-prone *Ldlr*^−/−^ recipient mice.^3,4^ Lethally irradiated *Ldlr*^−/−^ (CD45.1) mice were transplanted with bone marrow with hematopoietic-specific inactivation of *Tet2* (*Tet2*^fl/−^ *Vav1-Cre*^+^ CD45.2, or *Tet2* knockout [*Tet2*KO]) or WT (*Vav1-Cre^+^ CD45.2* or WT) control. Four weeks after transplantation, mice were fed WD for an additional 6 to 9 weeks^3^ (Figure 2A). Flow cytometry revealed that donor (CD45.2) cells repopulated recipient CD45.1 resident immune cells, including macrophages, accounting for >90% of cardiac immune cells in transplanted mice (Table S2). To assess for structural remodeling, we performed cardiac magnetic resonance imaging in the mice and did not find any significant differences in anatomical chamber volumes or ventricular function (Figure S3A). Histology was performed to assess interstitial fibrosis using Masson’s Trichrome staining, and again, there were no significant differences between donor genotypes (Figure S3B).

Surface ECG of mice showed no differences in typical parameters, including heart rate, PR interval, and QRS duration, between *Ldlr*^−/−^ mice with hematopoietic-specific inactivation of *Tet2* versus WT control (Table S3). We then performed in vivo invasive electrophysiology studies to assay sinus node function, atrioventricular node function, tissue characteristics, and arrhythmia inducibility, as previously described.^17,18,20^ There were no differences in sinus node recovery time, atrioventricular Wenckebach cycle length, atrioventricular 2:1 cycle length, atrioventricular nodal effective refractory period, or ventricular effective refractory period regardless of donor bone marrow (Table S3). However, compared with WT controls, there was a 2-fold increase in the percentage of mice with any AF among those with hematopoietic-specific inactivation of *Tet2* as well as a significant increase in AF inducibility (Figure 2A through 2D). There was also a shortening of the atrial effective refractory period (AERP; 29.8±9.3 ms for *Tet2*KO versus 17.9±5.2 ms for WT; *P*=0.034), which can contribute to increased electrical excitability and susceptibility for re-entry, enhancing arrhythmogenesis and AF (Figure 2E).^31,32^ We next performed ex vivo optical mapping to determine the action potential characteristics and conduction propagation.^17,20^ In keeping with the in vivo changes in AERP, the right atrial action potential duration (RA APD) was abbreviated in *Ldlr*^−/−^ mice with hematopoietic-specific inactivation of *Tet2* fed WD (23.31±3.40 ms) compared with WT controls (20±2.89 ms; *P*=0.034; Figure 2F). Finally, we demonstrated a significant, >3-fold increase in atrial expression of Nlrp3 in *Tet2*-deficient marrow recipients, suggesting “priming” of the inflammasome (Figure 2G).

Specific mutated genes carry variable risk for CVD and other disorders in CHIP.^3,23,25^ Mutations in *DMNT3A* were the most commonly identified CHIP mutation. We thus performed similar studies in *Ldlr*^−/−^ mice with hematopoietic-specific inactivation of *Dnmt3a* (*Dnmt3a*^fl/−^ *Vav1*-Cre^+^ CD45.2, or *Dnmt3a*KO), fed WD for 6 to 9 weeks. In keeping with genotype-specific associations in human studies from the UK Biobank, only a minority of mice were inducible for AF, and mice with hematopoietic-specific inactivation of *Dnmt3a*KO did not have elevated AF inducibility (Figure S4A through S4C). These findings further support that increased AF inducibility is associated with hematopoietic loss of *Tet2*, and not the commonly mutated gene, *Dnmt3a*.

Mutant clone size, as assessed by VAF, is associated with greater risk of atherosclerotic disease, HF, and other inflammatory disorders.^3,5,22,26,33–35^ To assess the effect of hematopoietic-specific inactivation of *Tet2* chimerism and clonal expansion, we generated chimeric mice by transplanting bone marrow consisting of 10% WT (CD45.2) and 90% WT (CD45.1) or 10% hematopoietic-specific inactivation of *Tet2* (CD45.2) and 90% WT (CD45.1) into lethally irradiated *Ldlr*^−/−^ (CD45.1) recipients and fed WD for 6 to 9 weeks (Figure S5A). Chimeric *Ldlr*^−/−^ mice with 10% hematopoietic-specific inactivation of *Tet2* (CD45.2) and 90% WT (CD45.1) showed greater *Tet2* clonal expansion compared with WT controls (36.7±11.34% and 11.4±7.69%, respectively; *P*<0.0001; Figure S5B). Overall, we observed no difference in AF inducibility or AERP between the chimeric *Ldlr*^−/−^ mice, likely related to the heterogeneity of *Tet2*-deficient hematopoietic clonal expansion and requirements for an inducible model of AF in mice (Figure S5C and S5D). Expansion of *Tet*-deficient hematopoietic cells inversely correlated with significant shortening of the RA APD (Figure S5E), mimicking the association between VAF and incident AF observed in human cohort studies and consistent with AF susceptibility with abbreviated AERP and APD.^9,31,36,37^ This is also consistent with murine models requiring significant alterations to elicit AF, and more pronounced phenotypes in mouse models of CHIP with loss of *Tet2* in hematopoietic lineages.^3,9,28,31,38^

### *Tet2*-Enhanced AF Requires Inflammasome Activation

*Tet2*-deficient hematopoietic cells have increased levels of proinflammatory cytokines such as IL-1β and IL-6, and humans with *TET2*-mutant CHIP have elevated levels of IL-1β.^3,4,15,39^ In mice, Nlrp3 inhibition, which results in decreased activation of IL-1β, attenuates hematopoietic *Tet2*KO-driven atherosclerosis, HF, liver fibrosis, and gout.^4,27,34,35^ As noted, lethally irradiated *Ldlr*^−/−^ mice with hematopoietic-specific inactivation of *Tet2* showed markedly higher atrial Nlrp3 expression compared with WT controls (Figure 2G). We noted a similar trend in other inflammasome complex components: apoptosis-associated speck-like protein containing a CARD (caspase recruitment domain; ASC), pro–caspase-1, and cleaved caspase-1/p20 (Figure S6A). Increased atrial Nlrp3 inflammasome activation has been shown to promote electrical remodeling and increase AF susceptibility in mice.^9,30^ To examine the necessity of the Nlrp3 inflammasome in atrial arrhythmogenesis in our model, we generated models of Nlrp3 inactivation through genetic ablation and pharmacological inhibition.

**Figure 3.**
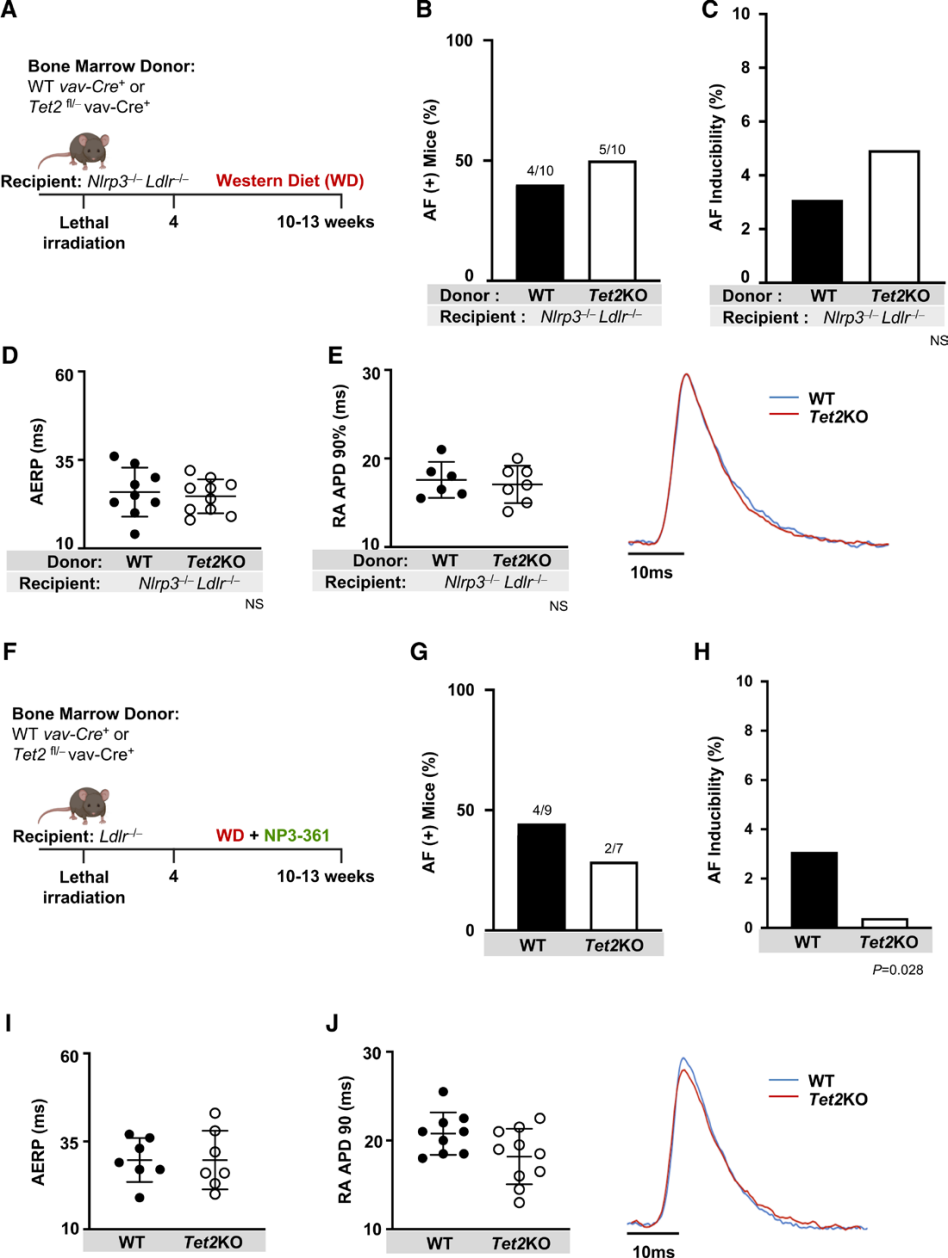
**NLRP3 inflammasome activation is required for enhanced AF inducibility stemming from hematopoietic-specific *Tet2* deficiency. A**, Lethally irradiated *Ldlr*^−/−^ *Nlrp3*^−/−^ mice were transplanted with bone marrow with hematopoietic-specific inactivation of *Tet2* (*Tet2*KO) or WT controls and then fed WD for a total of 6 to 9 weeks. **B**, Hematopoietic-specific *Tet2* inactivation did not affect number of mice with AF (number of mice with AF/total mice in the group) or **C**, AF inducibility in *Ldlr*^−/−^ *Nlrp3*^−/−^ mice. **D**, NLRP3 deficiency abrogated *Tet2*KO-related shortening in AERP as well as **E**, RA APD90. Representative tracing (**right**). **F**, Lethally irradiated *Ldlr*^−/−^ mice were transplanted with bone marrow with hematopoietic-specific inactivation of *Tet2* (*Tet2*KO) or WT controls and then fed WD with an incorporated NLRP3 inhibitor, NP3-361, for a total of 6 to 9 weeks. **G**, Hematopoietic-specific *Tet2* deficiency did not increase number of mice with AF, nor **H**, AF inducibility in *Ldlr*^−/−^ mice treated with NP3-361. **I**, NP3-361 treatment abrogated *Tet2*KO-related shortening in AERP as well as **J**, RA APD90. Representative tracing (**right**). Statistical testing: Fisher exact test for **B**, **C**, **G**, and **H**; 2-tailed Student *t* test and mean (SD) shown for **D**, **E**, **I**, and **J**. AERP indicates atrial effective refractory period; AF, atrial fibrillation; KO, knockout; LDLR, low-density lipoprotein receptor; NLRP3, NLR (NACHT, LRR [leucine rich repeat]) family pyrin domain containing protein 3; RA APD90, right atrial action potential duration at 90% repolarization; TET2, tet methylcystosine dioxygenase 2; and WD, Western diet; and WT, wild-type.

To determine the role of inflammasome activation in recipient cardiomyocytes, we assessed genetic inactivation of *Nlrp3* in our model. Recipient *Ldlr*^−/−^ *Nlrp3*^−/−^ mice were lethally irradiated and reconstituted with bone marrow with hematopoietic-specific inactivation of *Tet2* or WT control and fed WD for 6 to 9 weeks (Figure 3A). In contrast with the recipient *Ldlr*^−/−^ mice, recipient *Ldlr*^−/−^ *Nlrp3*^−/−^ mice were protected from *Tet2*KO-mediated differences in AERP, APD, or AF propensity (Figure 3B through 3E). These results demonstrate the necessity of recipient Nrlp3 in AF inducibility because of hematopoietic-specific inactivation of *Tet2* in *Ldlr*^−/−^ mice.

Several small molecules, including MCC950, have been shown to effectively inhibit NLRP3.^40^ We examined the effect of Nlrp3 inhibition in *Ldlr*^−/−^ mice with hematopoietic-specific inactivation of *Tet2* using a potent and optimized small molecule antagonist of NLRP3, NP3-361, dosed in WD chow. NP3-361 binds to the NACHT domain of the NLRP3 protein and locks it in an inactive conformation, thereby preventing NLRP3 inflammasome activation in a manner analogous to other described NLRP3 inhibitors such as MCC950/cytokine release inhibitory drug (CRID) 3^41^ (Figure S7A and S7B). In pharmacokinetic analyses, we demonstrated similar drug levels during WD feeding regardless of mouse recipient or donor genetic status (Figure S7C through S7F). The exposure levels were well above the mouse whole blood IC_50_ of LPS (lipopolysaccharide)/ATP-mediated IL-1β secretion of 261 nM, sufficient to block inflammasome activity (Figure S7B). AF occurred in the minority of mice treated with the antagonist, with reduced AF inducibility, comparable to the recipient *Ldlr*^−/−^ *Nlrp3*^−/−^ model. Treatment of lethally irradiated *Ldlr*^−/−^ mice with NP3-361 prevented the *Tet2*KO-mediated augmentation in AF susceptibility (Figure 3F through 3H). as well as associated shortening in RA APD and AERP (Figure 3I and 3J).

We next assessed the role of donor or hematopoietic Nlrp3 activity on the susceptibility to AF. Lethally irradiated *Ldlr*^−/−^ mice reconstituted with hematopoietic-specific inactivation of both *Tet2* and *Nrlp3* (*Tet2*^fl/-^ *Vav1-Cre*^+^ CD45.2, *Nlrp3*^−/−^, or double knockout) or *Nlrp3*^−/−^ alone were fed WD or WD with NP3-361 for 6 to 9 weeks (Figure S8A). There were similar rates of AF and AF inducibility between mice receiving *Nlrp3*^−/−^ and double knockout bone marrow, without significant differences in AERP or RA APD (Figure S8B through S8E). However, treating double knockout mice with NP3-361 significantly reduced AF inducibility (Figure S8C). Overall, these studies underscore the necessity of recipient Nrlp3 activation in AF propensity, whereas the role of donor Nrlp3 warrants additional investigation.

### Prolonged Exposure to Hematopoietic-Specific Inactivation of *Tet2* Promotes AF in Nonatherogenic Mice

**Figure 4.**
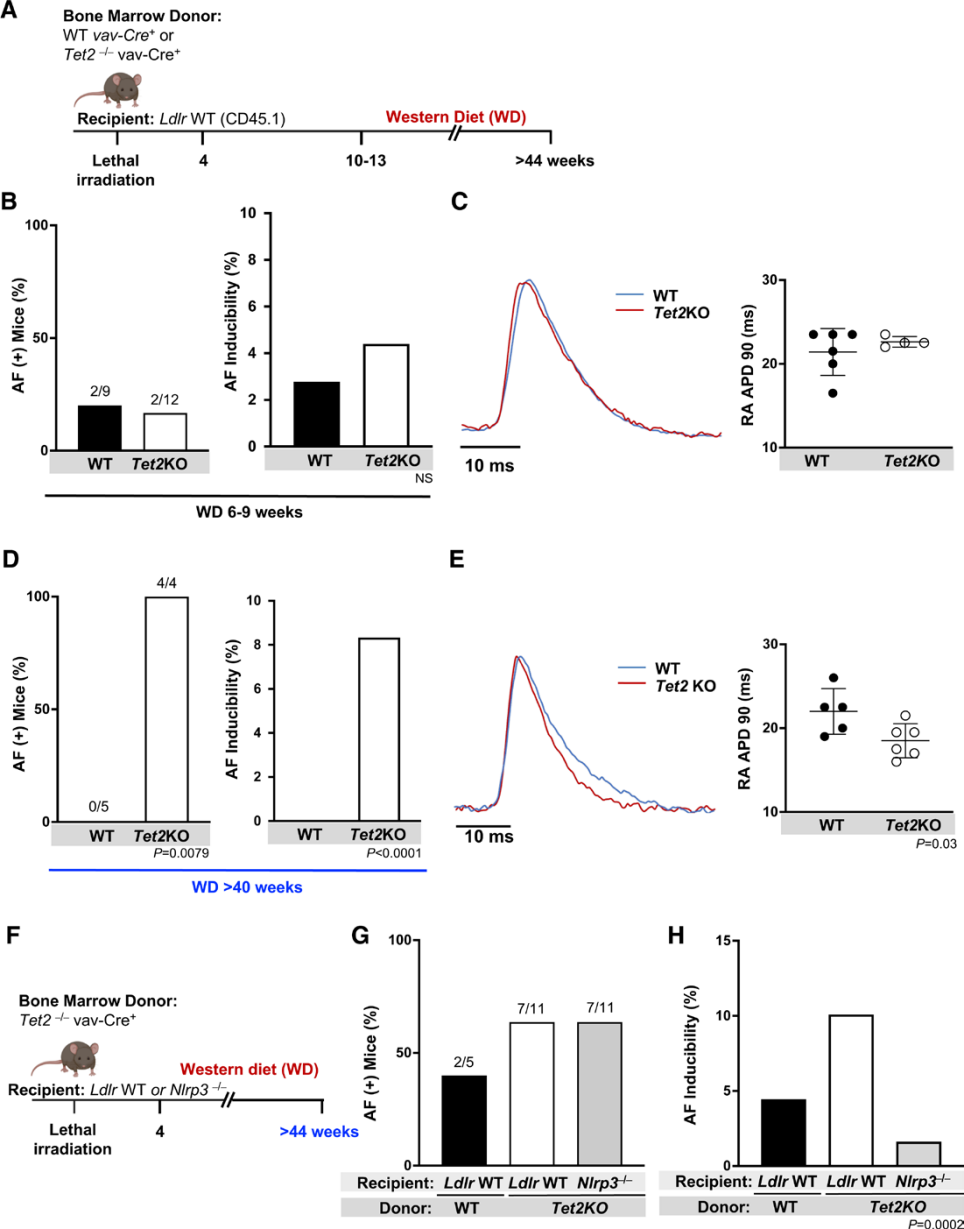
**Inflammasome activation is required for *Tet2*-enhanced AF inducibility in nonaccelerated atherogenic models. A**, Lethally irradiated *Ldlr* WT (CD45.1) mice were transplanted with bone marrow with hematopoietic-specific inactivation of *Tet2* (*Tet2*KO) or WT controls and fed WD. **B**, Hematopoietic-specific *Tet2* inactivation did not increase number of mice with AF (**left**; number of mice with AF/total mice in the group)or AF inducibility (**right**) in *Ldlr* WT mice. **C**, Hematopoietic-specific*Tet2* deficiency did not affect RA APD90 in *Ldlr* WT recipient mice after 6 to 9 weeks of WD. Representative tracing to the **left. D**, After feeding WD >40 weeks, AF was provoked in all hematopoietic-specific inactivated *Tet2* (*Tet2*KO) bone marrow recipients but none of the WT bone marrow recipients (**left**; number of mice with AF/total mice in the group), and there was a marked difference in AF inducibility (**right**). **E**, *Tet2*KO bone marrow recipients had a shorter RA APD90 compared with WT counterparts. Representative tracing to the **left. F**, Lethally irradiated *Ldlr* WT mice or *Nlrp3*^−/−^ mice were transplanted with bone marrow with hematopoietic-specific inactivation of *Tet2* (*Tet2*KO) or WT controls and then fed WD >40 weeks. **G**, There were no significant differences in number of mice with AF (number of mice with AF/total mice in the group); however, **H**, there was a significant difference in AF inducibility among the 3 groups. Statistical testing: Fisher exact test for **B** and **D**; 2-tailed Student *t* test and mean (SD) for **C** and **E**; χ^2^ test for **G** and **H**. AF indicates atrial fibrillation; KO, knockout; LDLR, low-density lipoprotein receptor; NLRP3, NLR (NACHT, LRR [leucine rich repeat]) family pyrin domain containing protein 3; RA APD90, right atrial action potential duration at 90% repolarization; TET2, tet methylcystosine dioxygenase 2; WD, Western diet; and WT, wild-type.

To account for any confounding from the atherogenic *Ldlr*^−/−^ model used thus far, we attempted to determine whether similar findings could be ascertained in a nonatherogenic mouse model. *Ldlr* WT (CD45.1) mice were lethally irradiated and transplanted with bone marrow from hematopoietic-specific inactivation of *Tet2* (*Tet2*KO) or WT controls. As with the *Ldlr*^−/−^ model, *Ldlr* WT mice were fed WD (Figure 4A). After 6 to 9 weeks of WD, there was no difference in AF inducibility between the 2 groups (Figure 4B), RA APD (Figure 4C), or atrial inflammasome protein expression (Figure S6B). Perhaps underlying this apparent discrepancy, *Ldlr* WT mice with hematopoietic *Tet2* deficiency at this age expressed significantly less atrial Nlrp3 protein than their *Ldlr*^−/−^ age-matched counterparts (Figure S6C). We noted increased AF susceptibility in *Ldlr* WT mice with hematopoietic *Tet2* deficiency emerged after >40 weeks of WD (Figure 4D). As with the *Ldlr*^−/−^ mice, increased AF inducibility was associated with shortening of the APD (Figure 4E). These data suggest a much slower effect of hematopoietic-specific inactivation of *Tet2* in *Ldlr* WT mice. However, this phenotype was again dependent on Nlrp3 activation, because genetic deficiency of *Nlrp3* prevented the AF inducibility caused by hematopoietic loss of *Tet2* (Figure 4F through 4H). Given the timeline required to generate the AF phenotype in *Ldlr* WT mice, we used the *Ldlr*^−/−^ mice for further downstream mechanistic analyses.

### Mice With Loss of *Tet2* in Hematopoietic Lineages Have Altered Cardiomyocyte Function

**Figure 5.**
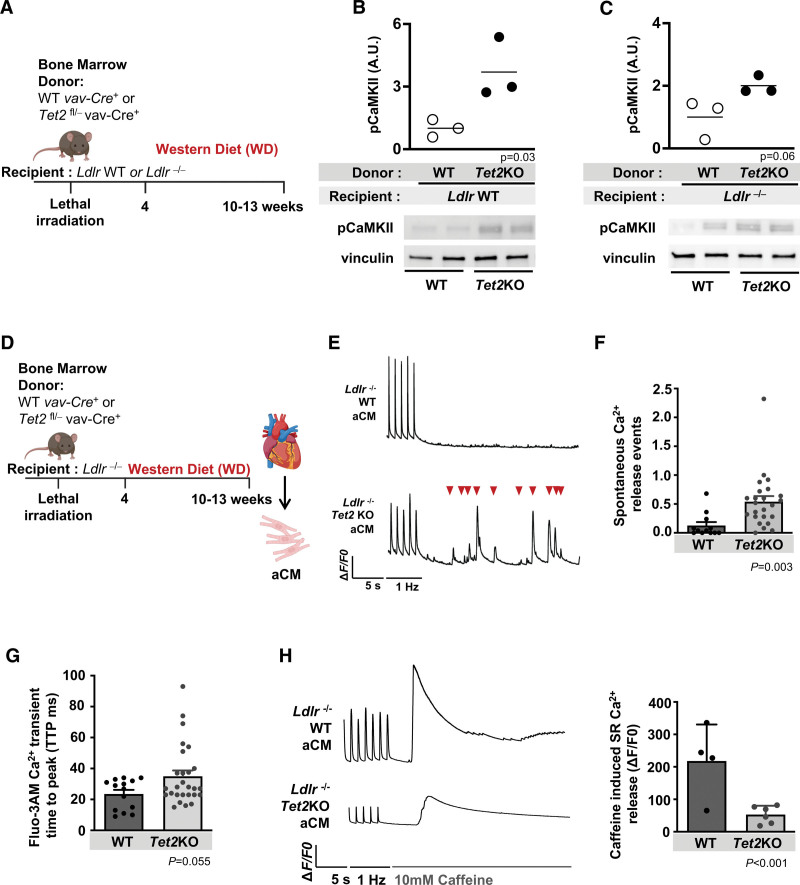
**Hematopoietic-specific inactivation of *Tet2* leads to altered cardiomyocyte sarcoplasmic reticulum calcium release. A**, Lethally irradiated *Ldlr* WT or *Ldlr*^−/−^ mice were transplanted with bone marrow with hematopoietic-specific inactivation of *Tet2* (*Tet2*KO) or WT controls and fed WD. **B**, Western blotting was performed to determine protein expression of phosphorylated calmodulin kinase II (CaMKII) in atrial tissue of *Ldlr* WT and **C**, *Ldlr*^−/−^ recipient mice. **D**, Lethally irradiated *Ldlr*^−/−^ mice transplanted with bone marrow with hematopoietic-specific inactivation of *Tet2* (*Tet2*KO) or WT controls were fed WD for 6 to 9 weeks. Atrial cardiomyocytes (aCM) were isolated and calcium studies performed using the calcium-sensitive dye Fluo-3 AM. **E**, Removal of pacing stimuli leads to spontaneous calcium release into cytosol (▼). **F**, Spontaneous calcium release events measured per second (data obtained from 2 mice per groups, n=12 events for WT and n=24 events for *Tet2*KO). **G**, Increased calcium transient amplitude measured during pacing (data obtained from 2 mice per groups, n=14 cells for WT and n=27 cells for *Tet2*KO). **H**, Removal of pacing stimuli and the addition of 10 mM caffeine leads to SR calcium emptying into the cytosol. Tracing to **left**, and quantification to **right**, showing reduced SR calcium release in *Tet2*KO aCMs (data obtained from 2 mice per group, n=4 cells for WT and n=6 cells for *Tet2*KO). △F/F0 is the change in fluorescence over baseline. Hz indicates the external pacing frequency stimuli. All Western blot quantification relative to loading control (vinculin) and then normalized. A.U., arbitrary units. Statistical tests: 2-tailed Student *t* test for all comparisons; linear mixed model for **F** through **H**. aCM indicates atrial cardiomyocte; KO, knockout; LDLR, low-density lipoprotein receptor; SR, sarcoplasmic reticulum; TET2, tet methylcystosine dioxygenase 2; WD, Western diet; and WT, wild-type.

Cytosolic calcium is regulated by CaMKII (Ca^2+^/calmodulin-dependent protein kinase II) and its phosphorylation of SR ryanodine receptor2 (RyR2) channels and SERCA2a (sarcoplasmic/endoplasmic reticulum Ca2^+^ ATPase 2a).^36,42–44^ Alterations in SR calcium handling can promote AF,^44–46^ and existing literature links inflammation and cytokines such as IL-1β with NLRP3 and CaMKII-dependent RyR2 phosphorylation, resulting in enhanced spontaneous calcium release events or even inducible AF.^37,42,47^ Given its central role in calcium handling, we were intrigued to find an upregulated expression of the phosphorylated, and hence, activated form of CaMKII in atrial tissue isolated from both *Ldlr* WT and *Ldlr*^−/−^ mice with hematopoietic-specific inactivation of *Tet2* (Figure 5A through 5C).

We next explored the effects of hematopoietic-specific inactivation of *Tet2* on atrial cardiomyocyte calcium handling in our mouse model. Atrial cardiomyocytes isolated from lethally irradiated *Ldlr*^−/−^ mice with hematopoietic-specific inactivation of *Tet2* or WT controls were incubated with a fluorescent calcium sensitive indicator, Fluo-3 AM, to visualize and quantitatively characterize calcium transients (Figure 5D), as previously described.^48,49^ Atrial cardiomyocytes from mice with hematopoietic-specific inactivation of *Tet2* had dysfunctional intracellular calcium handling resulting in a trend (*P*=0.055) toward a prolonged transient time to peak, and an increased frequency of spontaneous calcium release events after removal of depolarizing stimuli (Figure 5E through 5G). Release of calcium from the SR is predominantly mediated by RyR2; thus, SR calcium stores can provide an approximation of RyR2 activity. Caffeine stimulation was used to induce total calcium release and measure total SR calcium content. In the presence of 10 mM caffeine, cardiomyocytes isolated from *Ldlr*^−/−^ mice with hematopoietic-specific inactivation of *Tet2* showed a decrease in the SR calcium release compared with WT counterparts (Figure 5H). These results indicate that hematopoietic-specific loss of *Tet2* may affect cardiomyocyte RyR2 activity resulting in abnormal calcium release from the SR into the cytosol.^49,50^

**Figure 6.**
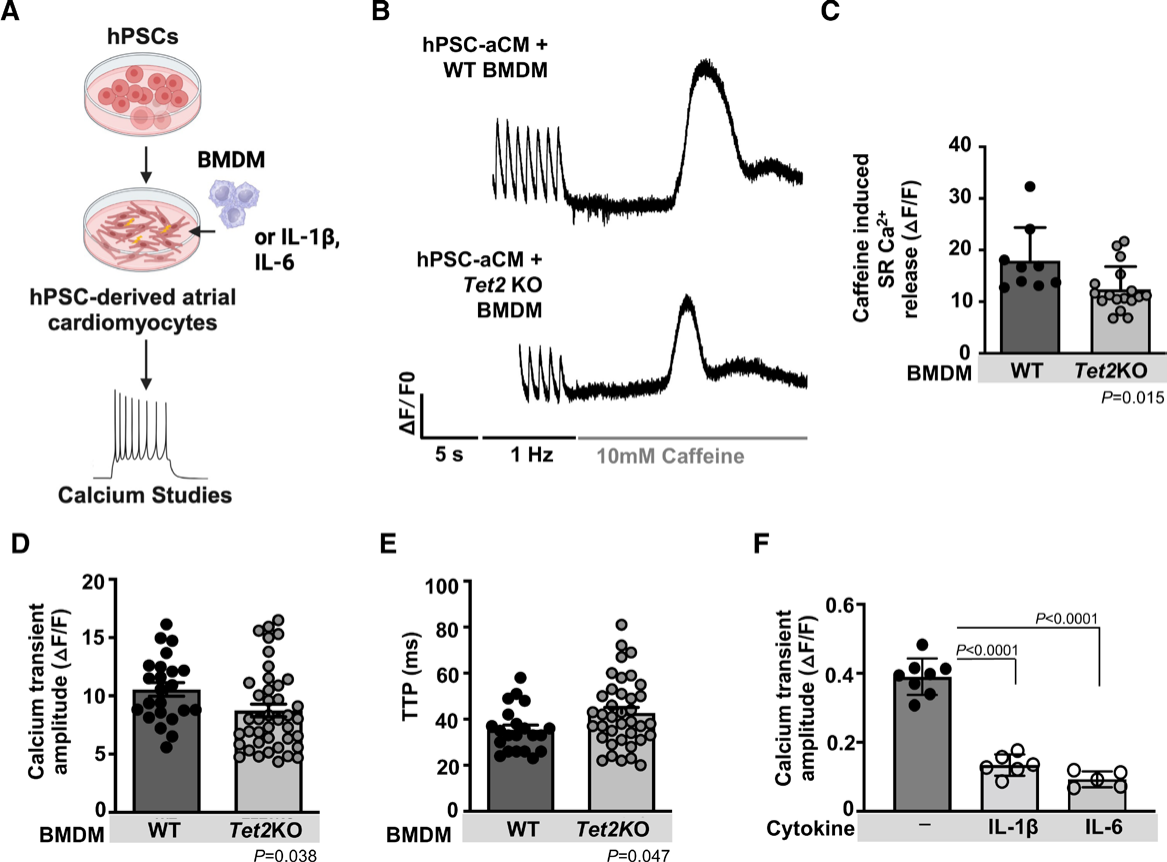
**Incubation with of *Tet2*-deficient bone marrow–derived macrophages alters cardiomyocyte calcium handling. A**, Human pluripotent stem cells differentiated to atrial cardiomyocytes (hPSC-aCM) were coincubated with *Tet2*-deficient (*Tet2*KO) bone marrow–derived macrophages (BMDM) or cytokines, IL-1β and IL-6. Calcium studies were performed using the calcium sensitive dye Fluo-3 AM. **B**, After 1 minute pacing at 1 Hz, fast perfusion of 10 mM caffeine leads to calcium emptying into the cytosol from SR. **C**, *Tet2*KO BMDM-treated hPSC-aCM showed reduced total SR Ca2^+^ release. △F/F0 is the change in fluorescence intensity over baseline. **D**, 1 Hz paced calcium transient amplitude (measured with Fluo-3 AM) is reduced in the hPSC-aCM incubated with *Tet2*KO BMDM compared with WT BMDM, with **E**, an increase in calcium transient time to peak (TTP). **F**, hPSC-aCM cardiomyocytes stimulated in culture with cytokines IL-1β 10 ng/mL or IL-6 10 ng/mL showed decreased SR calcium release (measured with Fluo-4 NW). Statistical tests: 2-tailed Student *t* test for **C** through **E**; 1-way ANOVA with Tukey multiple comparisons for **F**. IL-1β indicates interleukin 1β; IL-6, interleukin 6; SR, sarcoplasmic reticulum; TET2, tet methylcystosine dioxygenase 2; and WT, wild-type.

Previous studies have demonstrated the importance of cardiac macrophages affecting electrical conduction by facilitating AF in mice via cell-cell communication and possibly paracrine effects in cardiac conduction and AF susceptibility.^9,18,20,29,30,51^ We tested whether *Tet2*-deficient macrophages can alter cardiomyocyte function and calcium signaling. In an in vitro model, human pluripotent stem cell atrial cardiomyocytes coincubated with *Tet2*KO bone marrow–derived macrophages showed decreased total SR calcium release amplitude in the presence of caffeine, decreased calcium transient amplitude, and increased calcium transient time to peak when compared with human pluripotent stem cell atrial cardiomyocytes incubated with WT bone marrow–derived macrophages, consistent with our studies in isolated atrial murine cardiomyocytes (Figure 6A through 6E). We postulated that in these mice, chronic inflammation and increased release of cytokines such as IL-1β and IL-6 from macrophages may alter SR calcium release. Increased circulating proinflammatory cytokines, including IL-1β, correlate with AF progression in humans, promote arrhythmias in mice, and specifically depress cardiomyocyte function and cytosolic calcium release from the SR.^52–54^ We found that human pluripotent stem cell atrial cardiomyocytes stimulated with either human IL-1β or IL-6 had decreased calcium transient amplitude compared with untreated human pluripotent stem cell atrial cardiomyocytes (Figure 6F). These findings suggest that cytokines IL-1β and IL-6, known to be released from *Tet2*-deficient macrophages, may contribute to the dysfunctional calcium handling in our CHIP model.

## DISCUSSION

CHIP independently increases the risk of incident atherosclerosis and CAD, but the role of CHIP in arrhythmias, particularly AF, has remained less defined. Our findings indicate that individuals with CHIP, specifically those with large VAF *TET2* mutations, have an increased risk for incident AF. In both *Ldlr* WT and *Ldlr*^−/−^ mice fed a WD, hematopoietic-specific inactivation of *Tet2* led to increased AF inducibility. Mechanistically, our studies demonstrate the necessity of recipient Nlpr3 activity in AF propensity, through electrical, rather than structural, remodeling.

We found an association between CHIP and AF in the UK Biobank population, consistent with previous findings, and demonstrated that the risk of AF is dependent on the CHIP genotype.^55^ Individuals with mutations of *TET2* had augmented risk of developing AF relative to those with *DNMT3A* or CHIP mutations with smaller VAF size clones, recapitulated in our murine models. Growing evidence suggests chronic low-grade inflammation associated with aging contributes substantially to age-related disorders, including CVD and AF.^56–58^ It is notable that mice and humans with *TET2*-mutant CHIP have elevated IL-6 and IL-1β, which are key effectors of CHIP-mediated atherosclerosis and CVD.^3,15,22,59^ Previous studies highlight the importance of cardiomyocyte NLPR3 inflammasome activation and IL-1β in the pathogenesis of AF and atherosclerotic burden.^4,9,30,52–54^ We found that heightened susceptibility to AF in our mouse model depends on the Nlrp3 inflammasome, whose expression is markedly increased by hematopoietic-specific *Tet2* deficiency. In support of this finding, recipient *Nlrp3* deficiency abrogated the hematopoietic-specific *Tet2* deficiency–driven arrhythmogenesis in both atherogenic and nonatherogenic mouse models. We noted similar results in a study of NP3-361, a small-molecule NLRP3 inhibitor analogous to MCC950. However, because of its novelty, there is limited literature about this compound, and further work will be required before it can be considered for a translational role.

Our findings indicate that dysregulated cardiomyocyte calcium handling contributes mechanistically to the link between hematopoietic *Tet2* deficiency and AF. Hematopoietic-specific *Tet2* deficiency resulted in increased atrial expression of phosphorylated CaMKII, a central regulator of SR calcium flux. Atrial cardiomyocytes isolated from *Ldlr*^−/−^ mice with hematopoietic-specific inactivation of *Tet2* have abnormal calcium release from SR into the cytosol, demonstrating a role for a cell nonautonomous alteration in these cardiomyocytes. In vitro studies showed that *Tet2*-deficient macrophages coincubated with atrial cardiomyocytes can promote similar cardiomyocyte dysfunction, and this phenotype was also observed with the addition of IL-1β and IL-6 to atrial cardiomyocytes. Increased Nlrp3 inflammasome activation in cardiomyocytes is known to promote aberrant SR calcium release, promote electrical remodeling leading to shortening of atrial APD, and increase susceptibility for AF in mouse models.^8,9,30^ It is evident that further investigation is required to determine the molecular mechanisms that connect Nlrp3 activation, CaMKII, and SR calcium release, and how loss of *Tet2* in macrophages contributes to these alterations.

Although there is a growing burden of evidence about the role of inflammation in CVD, it has yet to affect mainstream clinical care. Colchicine, which may have some NLRP3 and IL-1β inhibitory effects, has shown efficacy in secondary prevention of cardiovascular events.^60^ Moreover, the role of IL-6 in CVD is being explored in ongoing trials of anti–IL-6 in patients with CAD and chronic kidney disease.^61^ In the CANTOS trial (Canakinumab Anti-Inflammatory Thrombosis Outcomes Study), patients with known CAD and above median hsCRP (high-sensitivity C-reactive protein) had decreased major adverse cardiac events and HF hospitalizations when treated with the neutralizing monoclonal anti–IL-1β canakinumab. Further post hoc analysis showed that individuals harboring *TET2* CHIP mutations derived enhanced benefit on canakinumab treatment compared with those without CHIP.^62^ These data warrant further investigation, because they suggest a role for targeted therapies on the basis of the identification of somatic mutations.

Contemporary strategies for the treatment of AF focus on symptom reduction through either rate or rhythm control strategies that aim to restore/maintain sinus rhythm. Currently available pharmacological therapies primarily act through modulation of ion channels and impart modest efficacy.^63^ There is growing evidence linking inflammation and AF, and targeting aberrant inflammation may provide a novel mechanistic approach to addressing contributing pathways in AF. Studies have shown resident cardiac macrophages can regulate cardiomyocyte function and modulate atrioventricular node conduction,^18^ IL-1β can affect electrical conduction and cardiomyocyte contractile function,^54^ and recruited and inflammatory cardiac macrophages can promote AF.^29^ Despite these findings, the use of anti-inflammatory agents in the prevention of recurrent AF in a postoperative or postprocedural event, including corticosteroids, anti–IL-1β, omega-3 fatty acids, and colchicine, have yielded mixed results.^64–67^ Similar to the CANTOS trial and subanalysis of individuals with CHIP, the presence of *TET2*-mutated CHIP could identify a more responsive patient population for the treatment of AF with specific NLRP3 inhibitors and the presence of *TET2* CHIP mutations as a biomarker. Recent studies identified carriers of hematopoietic somatic mosaicism to be at greater risk for postoperative AF than those without these mutations, providing further support.^55^

Our understanding of the mechanistic basis of AF derives largely through the lens of the cardiomyocyte, which is critical to impulse propagation and myocardial contraction.^36^ However, the heart is composed of many noncardiomyocyte cell populations that cooperate intricately to impart cardiovascular function as well as pathophysiology.^68,69^ There is growing clinical and preclinical data about the importance of noncardiomyocytes in AF pathogenesis.^29^ Here, in both in vivo and in vitro models, we have demonstrated the contribution of mutated immune cells to electrical remodeling, altered calcium dynamics, and AF. Beyond somatic mutations such as those related to CHIP, common variants have been shown to explain a majority of the heritable predisposition to AF, but most existing mechanistic studies have focused on their role in cardiomyocytes. A broader look at noncardiomyocyte cell types, both within and extrinsic to the heart, may further our continued understanding of AF pathogenesis.

In summary, our analysis of a large human cohort study revealed that individuals with CHIP, and specifically *TET2* CHIP, have increased risk of developing AF. In vivo and in vitro studies showed that hematopoietic-specific loss of *Tet2*-enhanced cardiomyocyte inflammasome activity and abnormal cardiomyocyte SR calcium handling may underlie associated AF pathogenesis. We demonstrated that an orally administered small-molecule NLPR3 inflammasome inhibitor, NP3-361, abrogates AF inducibility in *Ldlr*^−/−^ mice with hematopoietic-specific inactivation of *Tet2*. These findings raise the possibility of targeting the inflammasome for the treatment of individuals with TET2-specific CHIP mutations with AF.

## ARTICLE INFORMATION

### Acknowledgments

A.E.L. designed and supervised research studies, conducted experiments, analyzed data, and wrote the article. A.C.B. supervised research studies, conducted experiments, analyzed data, and assisted with the article. L.X. conducted experiments, analyzed data, and assisted with the article. A.E.L. and A.C.B. contributed equally as first authors. A.N. conducted research studies and analyzed data. J.Y., W.W., M.A., C.B.H., P.F.B., M.M.C., and V.S. conducted selected experiments. C.F. supervised research studies, analyzed data, and provided key reagents. A.B. conducted selected experiments and analyzed data. A.G.B., P.N., and D.M. supervised research. P.E., P.L, and B.L.E. supervised research, secured research funding, and contributed to writing of the article. The authors gratefully acknowledge Cecelia Castelano and Dylan Adams for experimental support, and Siddhartha Jaiswal, Jonathan M. Tsai, Adam S. Sperling, and Peter G. Miller for assistance and scientific discussion. Illustrations were made in BioRender (BioRender.com).

### Sources of Funding

This work was supported by the John S. LaDue memorial fellowship in cardiology, the American Society of Hematology research training grant for fellows, and the American College of Cardiology Merck fellowship in cardiovascular disease and the metabolic syndrome (A.E.L.). A.C.B. was supported by National Institutes of Health (NIH) grant T32HL007604. L.X. was supported by the American Heart Association (AHA; 20CDA35260081). A.N. received funding from the Knut and Alice Wallenberg Foundation (KAW 2017.0436) and the SciLifeLab & Wallenberg data driven life science program (KAW 2020.0239). W.J.W. was supported by an RUNX1 research program and Alex’s Lemonade Stand Foundation early career investigator grant. M.A. received research funding from the Deutsche Forschungsgemeinschaft (AG252/1-1). B.L.E. was supported by NIH (R01HL082945, P01CA066996, P50CA206963, and R35CA253125), the Howard Hughes Medical Institute, Novartis, and the Adelson Medical Research Foundation. A.E.L, B.L.E, A.G.B., and P.N. were supported by the Leducq Foundation transatlantic network on clonal hematopoiesis and atherosclerosis. A.G.B. receives funding support from the Burroughs Wellcome Foundation career award for medical scientists and the director of NIH early independence award (DP5 OD029586). P.N. receives funding support from NIH (R01HL142711, R01HL148050, R01HL151283, and R01HL148565), Fondation Leducq, and Massachusetts General Hospital (Hassenfeld research scholar). P.L. receives funding support from the National Heart, Lung, and Blood Institute (1R01HL134892 and 1R01HL163099-01), AHA (18CSA34080399), the RRM charitable fund, and the Simard fund. P.T.E. was supported by grants from NIH (1RO1HL092577, 1R01HL157635, and 5R01HL139731), AHA (18SFRN34230127 and 961045), and the European Union (MAESTRIA 965286).

### Disclosures

B.L.E. has received research funding from Celgene, Deerfield, Novartis, and Calico and consulting fees from GRAIL. He is a member of the scientific advisory board and shareholder for Neomorph, TenSixteen Bio, Skyhawk Therapeutics, and Exo Therapeutics. P.T.E. receives sponsored research support from Bayer AG, IBM Research, Bristol Myers Squibb, Pfizer, and Novo Nordisk; he has also served on advisory boards or consulted for MyoKardia and Bayer AG. P.L. has received research funding from Novartis, Novo Nordisk, and Genentech, and is an unpaid consultant to, or involved in clinical trials for, Amgen, AstraZeneca, Baim Institute, Beren Therapeutics, Esperion Therapeutics, Genentech, Kancera, Kowa Pharmaceuticals, Medimmune, Merck, Moderna, Novo Nordisk, Novartis, Pfizer, and Sanofi-Regeneron. P.L. is a member of the scientific advisory boards for Amgen, Caristo Diagnostics, Cartesian Therapeutics, CSL Behring, DalCor Pharmaceuticals, Dewpoint Therapeutics, Eulicid Bioimaging, Kancera, Kowa Pharmaceuticals, Olatec Therapeutics, Medimmune, Novartis, PlaqueTec, TenSixteen Bio, Soley Therapeutics, and XBiotech, Inc. P.N. has received research funding from Amgen, Apple, Boston Scientific, and Novartis, and has received consulting income from Apple, Blackstone Life Sciences, Novartis, Foresite Labs, and Genentech; he is a member of the scientific advisory board and a shareholder of TenSixteen Bio and has spousal employment at Vertex, all unrelated to the present work. A.G.B. is a paid consultant for Foresite Labs and is member of the scientific advisory board and a shareholder of TenSixteen Bio. D.M. is the chief scientific officer of the Leducq Foundation, unrelated to the present work. A.B. has received research funding from Thryv Therapeutics and has received consulting income from Cardiologs, all unrelated to the present work. M.A. received consulting fees from German Accelerator Life Sciences and is a cofounder of, and holds equity in, iuvando Health, all unrelated to the present work. A.E.L. is a former member of TenSixteen Bio, unrelated to the present work.

### Supplemental Material

Expanded Methods

Figures S1–S8

Tables S1–S3

Reference 70

## Supplementary Material


