# Supplementary material for: Clonal Hematopoiesis of Indeterminate Potential With Loss of Tet2 Enhances Risk for Atrial Fibrillation Through Nlrp3 Inflammasome Activation
Source: Circulation. 2024 Feb 15;149(18):1419–34. doi: 10.1161/CIRCULATIONAHA.123.065597 (PMC11058018; doi:10.1161/CIRCULATIONAHA.123.065597)
Supplement: Supplementary file 1 [file cir-149-1419-s001.pdf]

Clonal hematopoiesis of indeterminate potential with loss of *Tet2* enhances risk for atrial fibrillation through *Nlrp3* inflammasome activation

Supplemental Materials

Expanded Methods

Figures S1-8

Supplemental Tables S1-3

Reference 70

Expanded Methods

Mouse whole blood assay

Mouse whole blood was collected in Li-Heparin tubes from C57BL/6J female mice, and diluted 1:1 in RPMI 1640+Glutamaxx (Gibco). Compound NP3-361 was diluted 1/3 in 90% DMSO (starting concentration 10mM) in 384-well plates resulting in a 12-point dilution titration and transferred into an empty assay plate using an ECHO555 (LabCyte). 40 mL of 50% mouse blood was added to wells. After a 60-minute incubation at 37°C, 5 mL of LPS crude (Sigma, L4391 10mg/mL diluted in RPMI 1640+Glutamaxx) was added and incubated for 4 hours before addition of 1mL ATP (Sigma A2383, 150mM in PBS, pH7.2). After 30 min incubation at 37°C, plates were centrifuged (1000 x g, 5min), and 10μL of supernatant were transferred to an assay plate and IL-1β was detected by HTRF (CisBio) following manufacturers' instructions. IC_{50} values were calculated from the plot of percentage of inhibition vs the inhibitor concentration by a logistics fit according to: $[y = A2 + (A_1 – A_2)/ (1 + (x/ IC_{50})^{\wedge} p)]$ where y is the %-inhibition at

the inhibitor concentration, x. A_1 is the lowest inhibition value, i.e. 0 %, and A_2 the maximum inhibition value, i.e. 100 %. The exponent, p, is the Hill coefficient.

Flow cytometry

Peripheral blood was obtained by retro-orbital bleed into EDTA collection tubes. Red blood cells were lysed (ACK lysis buffer) from whole blood prior to flow cytometry analysis. Cell populations were analyzed using FACSCANTO II (BD). The antibodies (BioLegend) used for cell surface staining include : FITC anti-mouse CD45.1 (Ly5.1), PE anti-CD45.2, (104), PerCP/Cyanine5.5 anti-mouse CD3 (17A2), APC/Cyanine5.5 anti-mouse CD11b (M1/70), PacBlue anti-mouse Gr-1 (RB6-8C5), BV510 anti-mouse B220 (RA3-6B2), BV510 anti-mouse CD45.1 (A20), PacBlue anti-mouse/human CD11b (M1/70), PerCP/Cyanine 5.5 anti-mouse Ly-6G (1A8), APC-Cyanine7 anti-mouse Ly-6C (RB6-8C5), PeCy7 CD11c (N418), FITC anti-mouse F4/80 (BM8). Cells were stained for 30 min at 4C in 2% FBS/PBS (vol/vol) buffer and flow cytometry was performed using the BD FACSCantoII system and analyzed on FlowJo software.

Cardiac MRI

MRI was performed with a 4.7-Tesla horizontal bore Pharmascan system (Bruker) equipped with a custom-built mouse cardiac coil (Rapid Biomedical) to obtain cine images of the left atrial and ventricular short axes. ECG and respiratory gating (SA Instruments), a gradient echo FLASH-sequence and a dedicated mouse cardiac volume coil in a bird cage design were used. Imaging parameters were as follows: time to echo (TE), 2.7 to 5 ms; 16 frames per RR interval (TR 7.0-15 ms depending on heart rate); in-plane resolution, 150x150 µm; slice thickness, 1 mm; NEX 4;

flip angle, 30 degrees. Anatomical and functional parameters were quantified using Horos software.

Bone marrow isolation, transplantation, and bone marrow derived macrophages

Recipient mice (CD45.1) aged 6-8 weeks were lethally irradiated with two doses of gamma-radiation (475cGy) at 3-hour intervals. Whole bone marrow was obtained from donor CD45.2 appropriate floxed mice with WT *Vav1-Cre*$^{+}$ controls. Isolated whole bone marrow cells were transplanted at $2x10^{6}$ cells per mouse using a retro-orbital intra-arterial approach. 4-5 weeks post-transplantation, confirmation of bone marrow reconstitution was evaluated by flow cytometry for CD45.2^{+} donor peripheral blood cells. Reconstituted mice were fed WD for a minimum of 5 weeks. For *in vitro* bone marrow derived macrophage (BMDM) differentiation, isolated whole bone marrow cells were plated at 350,000 cells/cm^{2} in Iscoves Media (Gibco) and 25ng/ml GM-CSF (Miltenyi) for 7 days. BMDMs were plated at $1x10^{6}$ cells/ml and incubated with cardiomyocytes and human IL-1β or IL-6 (InvivoGen) for 24 hours prior to calcium imaging studies.

Immunoblotting

Snap frozen atrial tissue samples were lysed using a Tissue Lyser (Qiagen) in protein lysis buffer supplemented with protease and phosphatase inhibitors (Boston BioProducts) and protein content was measured using a commercially available kit (DC Protein Assay, Bio-Rad). Equal amounts (15-25 mg) were treated with Laemmli buffer and β-mercaptoethanol and incubated at 100°C for 5 minutes. Lysate were electrophoresed on 4-20% SHS-polyacrylamide resolving gel and transferred to a PVDF membrane. The membrane was incubated overnight at 4° C with relevant

antibodies (NLRP3 (Invitrogen, 25N10E9), ASC (Santa Cruz, sc-514414), Caspase1(Santa Cruz, sc-56036), phosphoylated CamKII (Abcam 182647), vinculin (Sigma-Aldrich, V9264)), and then development was completed with secondary antibody for 2 hours at room temperature. Antibody signal detection was achieved by employing the Clarity Western ECL Substrate (BioRad #1705061). All protein quantification is relative to loading control (vinculin) and then normalized. A.U., arbitrary units. Imaging and image quantification were done via BioRad Chemidoc Touch Imaging System and ImageLab, respectively.

Histological analysis

Hearts were perfused with PBS prior to isolation, fixed in 4% paraformaldehyde (PFA) overnight at 4°C then embedded in paraffin. Hematoxylin-eosin (H+E) and Masson's Trichrome staining were performed using standard protocols according to manufacturer's instructions (MilliporeSigma). Histology core labs include Brigham and Women's Hospital Histo-Pathology Core, Massachusetts General Hospital Histology core and iHisto Labs.

Cardiomyocyte isolation

Atrial cardiomyocytes were isolated from adult mice using standard protocols[17,20] , where hearts were excised from ketamine/xylazine anesthetized mice, cannulated and perfused at 37°C at 3ml/min with calcium free buffer and digested with collagenase II. Hearts were manually dissected, and isolated cardiomyocytes were seeded on laminin coated plates in M199 medium (supplemented with 5% fetal bovine calf serum and 2,3 butanedione monoxime) for 1 hour.

Cardiomyocyte stem cell differentiation

Human pluripotent stem cells (hPSC, WA07 [H7], WiCell), were maintained in Essential 8 Medium on Geltrex extracellular matrix (Invitrogen) using feeder-free conditions. The hPSCs were then maintained in RPMI 1640/B-27 without insulin (Invitrogen) with Geltrex (1:100) and stimulated over 24 hours with CHIR00921 (10uM; Stemgent). Supplementation with retinoic acid (ATRA 1 μM) was used for atrial differentiation. Differentiation and maintenance of cells was performed as previously described with minor modifications for atrial differentiation.[70] hPSC atrial cardiomyocytes (hPSC-aCM) were incubated with human recombinant IL-1β and IL-6 (Miltenyi Biotech), at 10ng/ml for 24 hours.

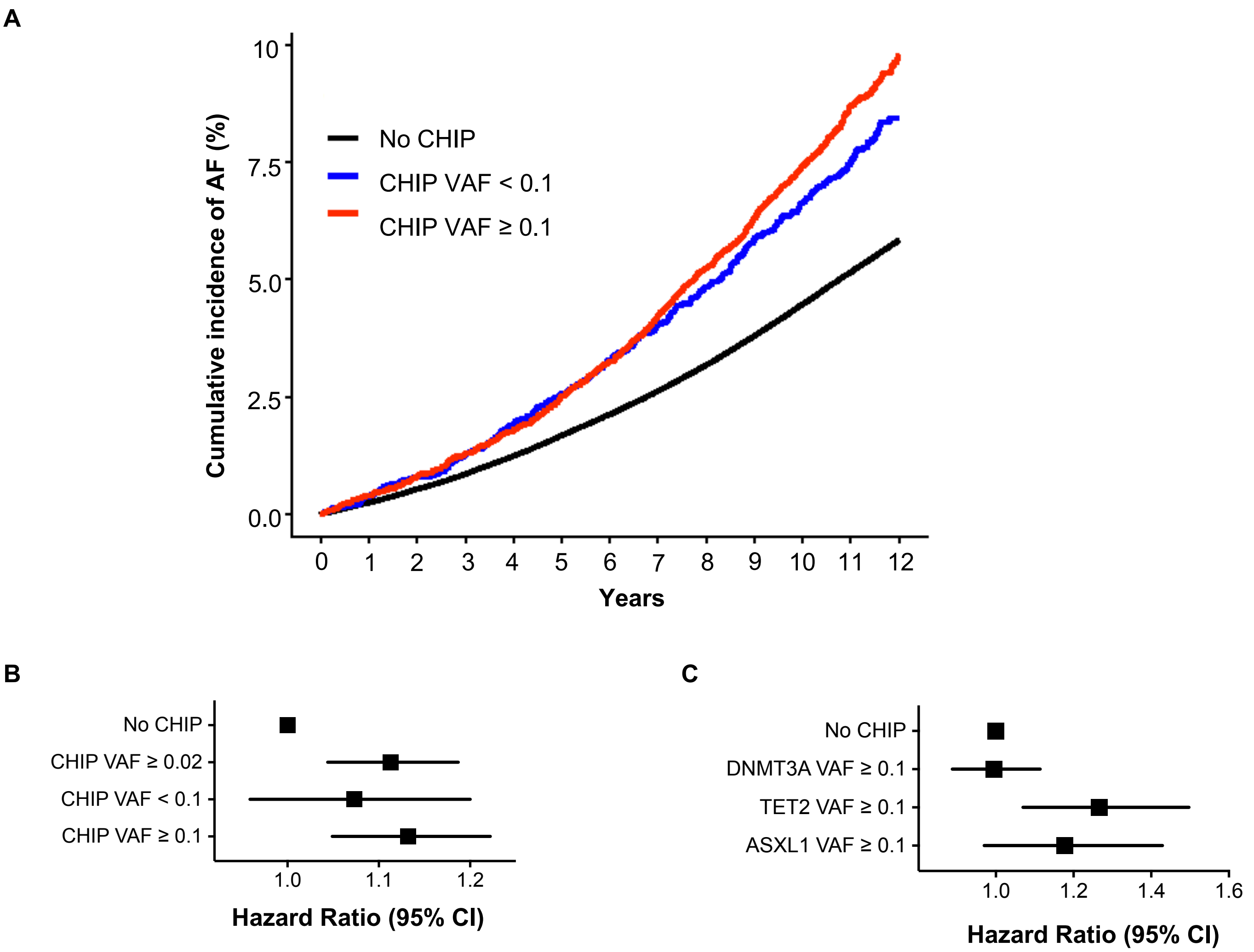

	**Group**	**AF**	**HR (95% CI)**	***P***
No CHIP	345513	17620		
CHIP VAF ≥ 0.02	12584	1000	1.113 (1.044, 1.187)	0.00101
CHIP VAF < 0.1	4223	311	1.073 (0.959, 1.2)	0.22
CHIP VAF ≥ 0.1	8361	689	1.132 (1.049, 1.222)	0.00142

	**Group**	**AF**	**HR (95% CI)**	***P***
No CHIP	345513	17620		
DNMT3A VAF ≥ 0.1	4695	324	1.02 (0.914, 1.139)	0.781
TET2 VAF ≥ 0.1	1433	143	1.27 (1.077, 1.498)	0.00442
ASXL1 VAF ≥ 0.1	997	107	1.176 (0.972, 1.422)	0.0956

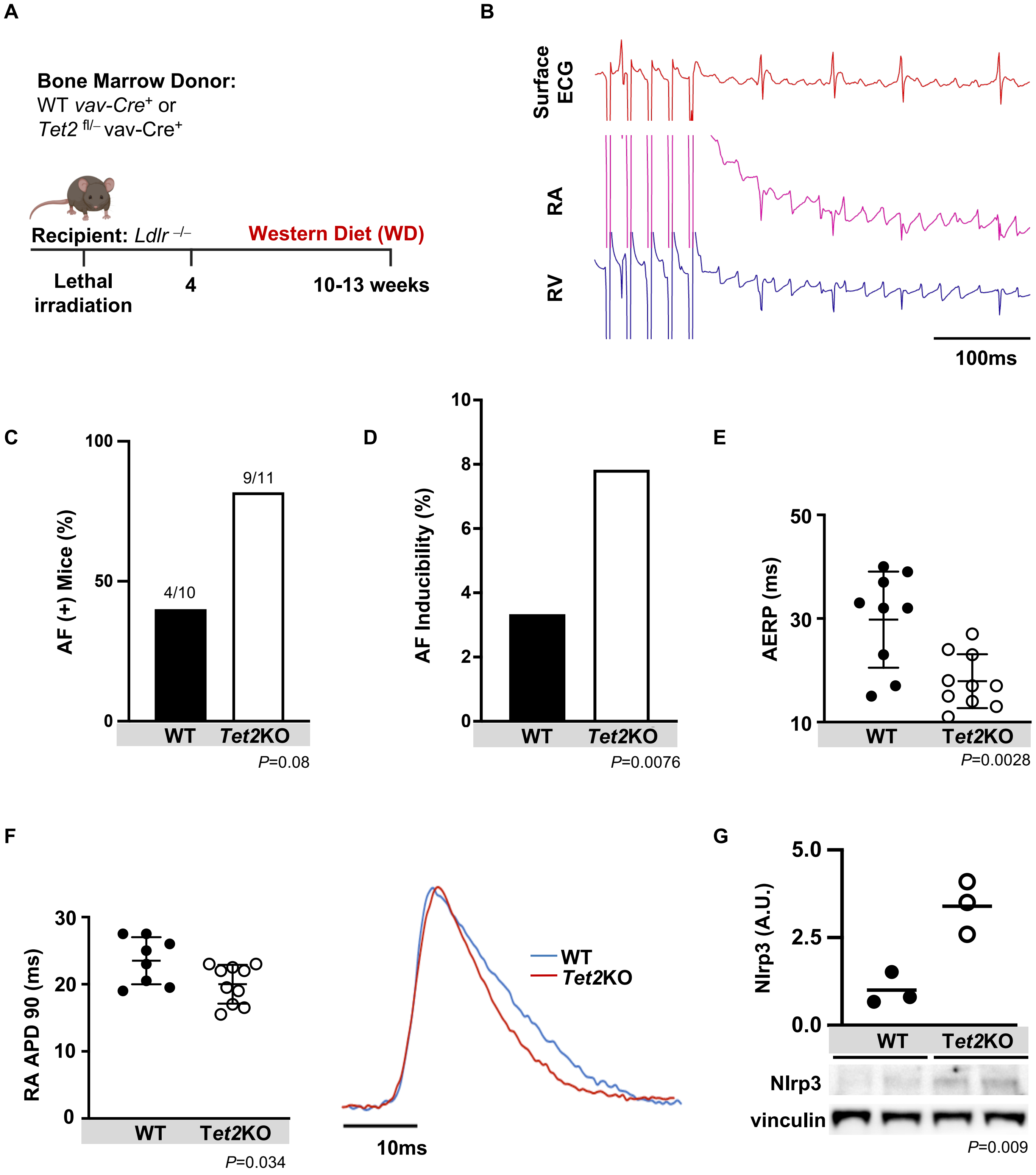

A
Bone Marrow Donor:
WT vav-Cre+ or
Tet2 fl/– vav-Cre+
Recipient: Ldlr –/–
Western Diet (WD)
Lethal irradiation
4
10-13 weeks
B
Surface ECG
RA
RV
100ms
C
AF (+) Mice (%)
4/10
9/11
WT
Tet2KO
P=0.08
D
AF Inducibility (%)
WT
Tet2KO
P=0.0076
E
AERP (ms)
WT
Tet2KO
P=0.0028
F
RA APD 90 (ms)
WT
Tet2KO
P=0.034
WT
Tet2KO
10ms
G
Nlrp3 (A.U.)
WT
Tet2KO
Nlrp3
vinculin
P=0.009

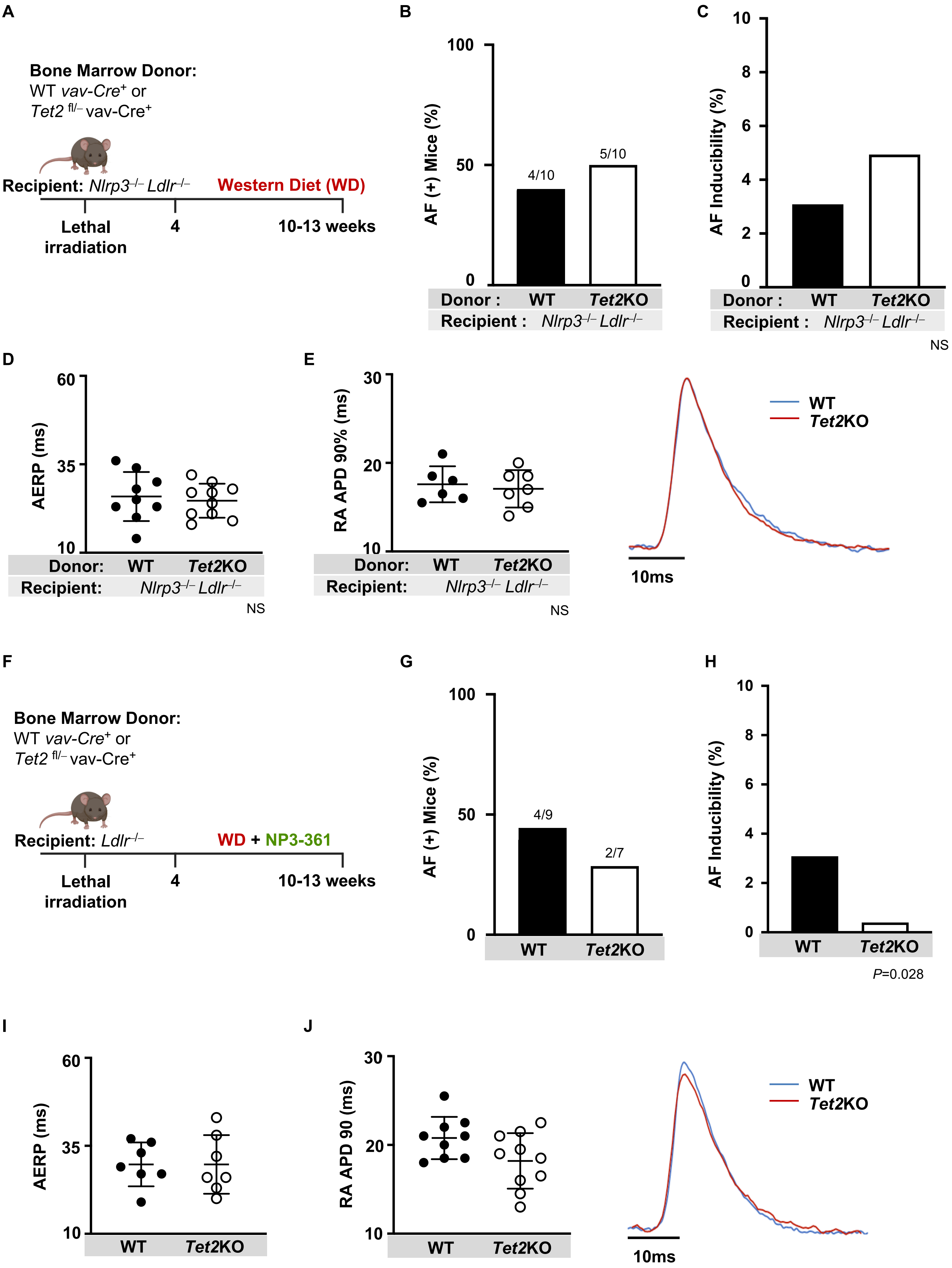
A
Bone Marrow Donor:
WT vav-Cre+ or
Tet2 fl/– vav-Cre+
Recipient: Nlrp3–/– Ldlr–/–
Western Diet (WD)
Lethal irradiation
4
10-13 weeks
B
AF (+) Mice (%)
100
50
0
4/10
5/10
Donor : WT Tet2KO
Recipient : Nlrp3–/– Ldlr–/–
C
AF Inducibility (%)
10
8
6
4
2
0
Donor : WT Tet2KO
Recipient : Nlrp3–/– Ldlr–/–
NS
D
AERP (ms)
60
35
10
Donor: WT Tet2KO
Recipient: Nlrp3–/– Ldlr–/–
NS
E
RA APD 90% (ms)
30
20
10
Donor: WT Tet2KO
Recipient: Nlrp3–/– Ldlr–/–
NS
WT
Tet2KO
10ms
F
Bone Marrow Donor:
WT vav-Cre+ or
Tet2 fl/– vav-Cre+
Recipient: Ldlr–/–
WD + NP3-361
Lethal irradiation
4
10-13 weeks
G
AF (+) Mice (%)
100
50
0
4/9
2/7
WT Tet2KO
H
AF Inducibility (%)
10
8
6
4
2
0
WT Tet2KO
P=0.028
I
AERP (ms)
60
35
10
WT Tet2KO
J
RA APD 90 (ms)
30
20
10
WT Tet2KO
WT
Tet2KO
10ms

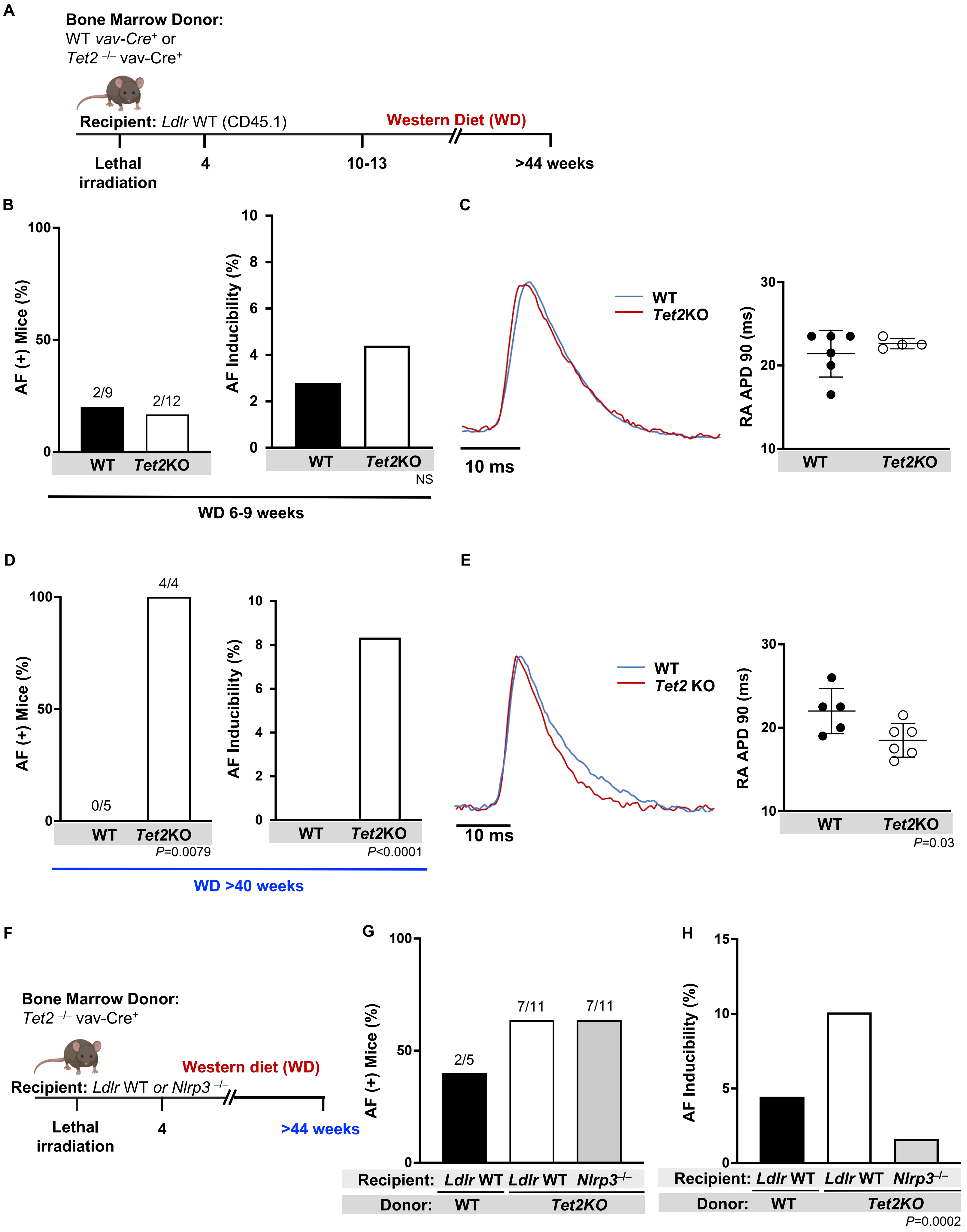
A
Bone Marrow Donor:
WT vav-Cre+ or
Tet2 −/− vav-Cre+
Recipient: Ldlr WT (CD45.1)
Western Diet (WD)
Lethal irradiation
4
10-13
>44 weeks
B
AF (+) Mice (%)
2/9
2/12
WT
Tet2KO
AF Inducibility (%)
NS
WD 6-9 weeks
C
WT
Tet2KO
10 ms
RA APD 90 (ms)
D
AF (+) Mice (%)
0/5
4/4
P=0.0079
AF Inducibility (%)
P<0.0001
WD >40 weeks
E
WT
Tet2 KO
10 ms
RA APD 90 (ms)
P=0.03
F
Bone Marrow Donor:
Tet2 −/− vav-Cre+
Western diet (WD)
Recipient: Ldlr WT or Nlrp3 −/−
Lethal irradiation
4
>44 weeks
G
AF (+) Mice (%)
2/5
7/11
7/11
Recipient: Ldlr WT Ldlr WT Nlrp3−/−
Donor: WT Tet2KO
H
AF Inducibility (%)
Recipient: Ldlr WT Ldlr WT Nlrp3−/−
Donor: WT Tet2KO
P=0.0002

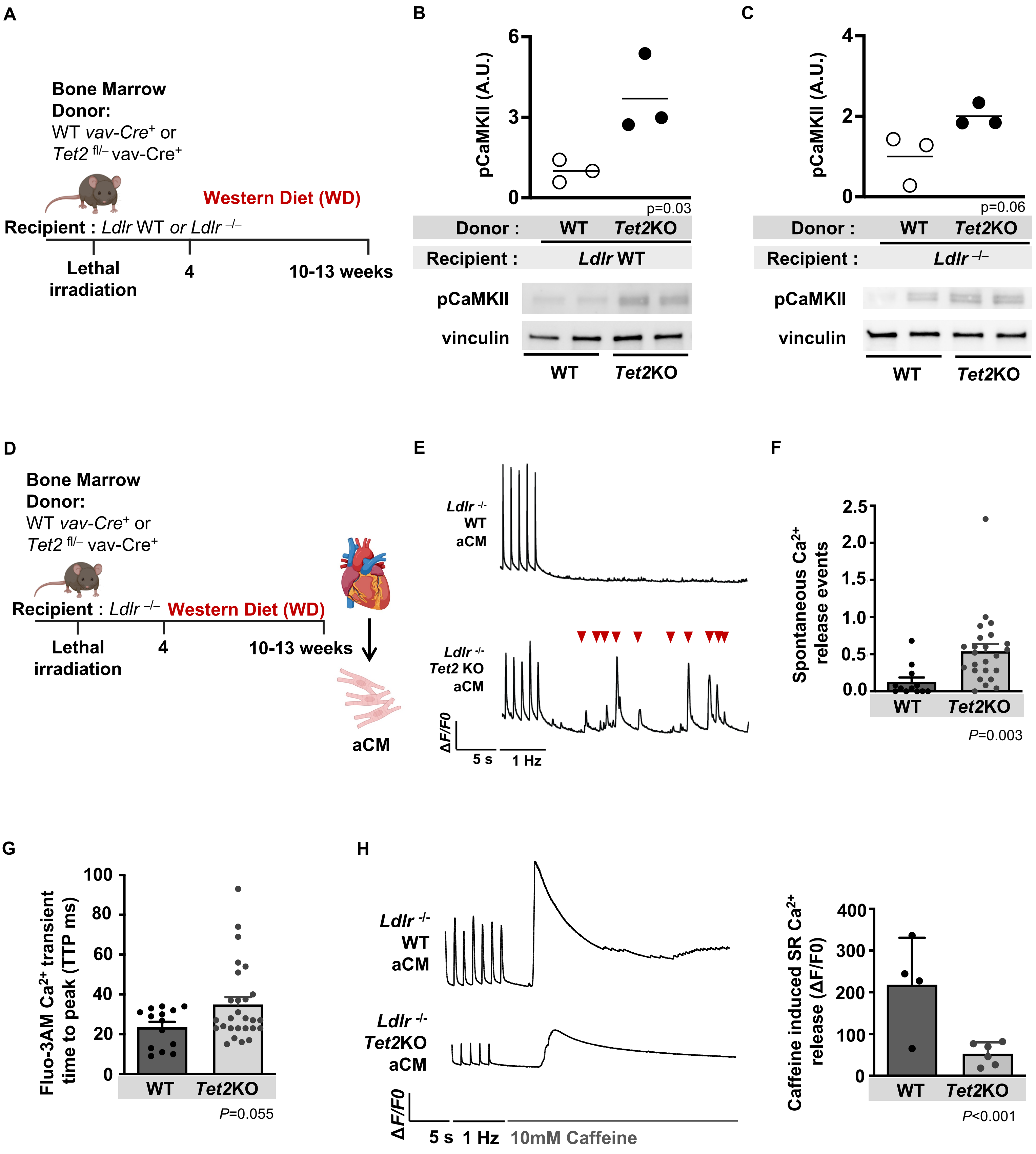

A
Bone Marrow Donor:
WT vav-Cre+ or
Tet2 fl/– vav-Cre+
Western Diet (WD)
Recipient : Ldlr WT or Ldlr –/–
Lethal irradiation
4
10-13 weeks
B
pCaMKII (A.U.)
p=0.03
Donor : WT Tet2KO
Recipient : Ldlr WT
pCaMKII
vinculin
WT Tet2KO
C
pCaMKII (A.U.)
p=0.06
Donor : WT Tet2KO
Recipient : Ldlr –/–
pCaMKII
vinculin
WT Tet2KO
D
Bone Marrow Donor:
WT vav-Cre+ or
Tet2 fl/– vav-Cre+
Recipient : Ldlr –/– Western Diet (WD)
Lethal irradiation
4
10-13 weeks
aCM
E
Ldlr -/- WT aCM
Ldlr -/- Tet2 KO aCM
ΔF/F0
5 s
1 Hz
F
Spontaneous Ca2+ release events
WT Tet2KO
P=0.003
G
Fluo-3AM Ca2+ transient time to peak (TTP ms)
WT Tet2KO
P=0.055
H
Ldlr -/- WT aCM
Ldlr -/- Tet2KO aCM
ΔF/F0
5 s 1 Hz 10mM Caffeine
Caffeine induced SR Ca2+ release (ΔF/F0)
WT Tet2KO
P<0.001

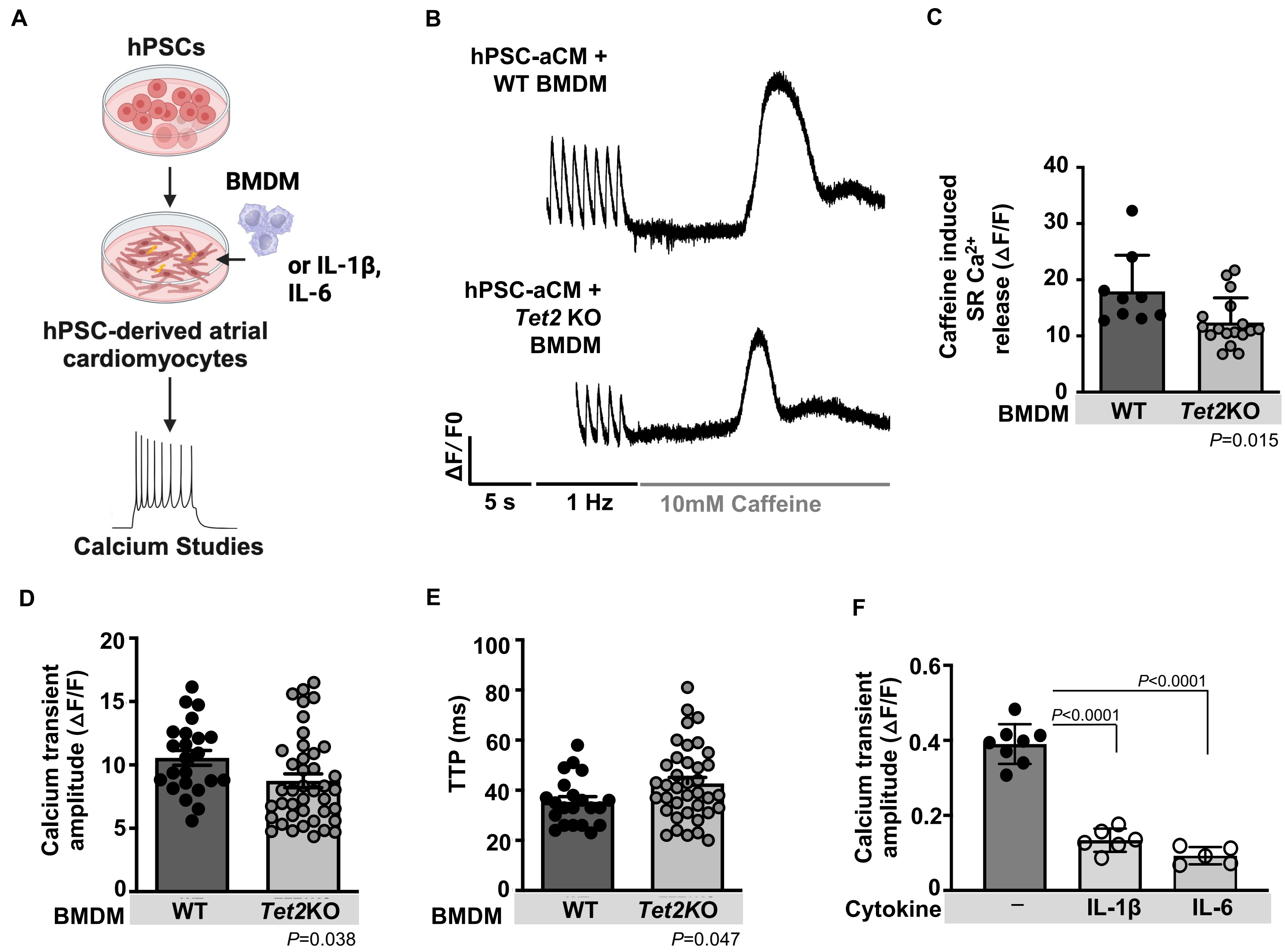
A
hPSCs
BMDM
or IL-1β, IL-6
hPSC-derived atrial cardiomyocytes
Calcium Studies
B
hPSC-aCM + WT BMDM
hPSC-aCM + Tet2 KO BMDM
ΔF/ F0
5 s
1 Hz
10mM Caffeine
C
Caffeine induced SR Ca2+ release (ΔF/F)
40
30
20
10
0
BMDM
WT
Tet2KO
P=0.015
D
Calcium transient amplitude (ΔF/F)
20
15
10
5
0
BMDM
WT
Tet2KO
P=0.038
E
TTP (ms)
100
80
60
40
20
0
BMDM
WT
Tet2KO
P=0.047
F
Calcium transient amplitude (ΔF/F)
0.6
0.4
0.2
0
P<0.0001
P<0.0001
Cytokine
–
IL-1β
IL-6

Name	ICD-10	ICD-9	OPSC-4
**AF/Aflutter**	I48, I48.0, I48.1, I48.2 I48.3 I48.4 I48.9	427.31, 427.32	
**Ablation**			K57.1, K62.1, K62.2, K62.3, K62.4
**Heart Failure**	398.91, 428, 428.1, 428.2, 428.3, 428.4, 428.9	I09.81, I50, I50.1, I50.2, I50.3, I50.4, I50.8, I50.9	
**Mitral stenosis**	134.0, 105.0, 105.1, 105.2, 105.8, 108, 108.8, 108.9	424.0, 394.0, 394.1, 394.2, 394.9, 396.1, 396.2, 396.3, 396.8, 396.9	
**Myeloproliferative neoplasm (MPN)**	D45, D46.9, D471.1, D47.3	207.1, 238.5 238.6, 238.7	
**Myeloid leukemia**	C92, C92.0, C92.1, C92.3, C92.4, C92.5, C92.7, C92.9	202.8, 203, 204.1 205.1, 205.2, 205.3, 205.8, 205.9, V10.6	

Supplemental Table 1. ***International Classification of Diseases, Ninth and 10th Revisions (*ICD-9, ICD-10) *codes*** **and Office of Population Censuses and Surveys Classification of Interventions and Procedures-4 (OPCS-4) codes in UK Biobank.**

	WT	*Tet2*KO	*P*
**CD45.1**	955	689	0.22
**CD45.2**	12826	12355	0.81
**CD45.2$^+$ neutrophil**	388	230	0.06
**CD45.2$^+$ macrophage**	5817	6421	0.68
**CD45.2$^+$ monocyte**	321	185	0.067

Supplemental Table 2. Donor hematopoietic stem cells re-populate the atria.
Lethally irradiated *Ldlr*$^{-/-}$ mice transplanted with WT or hematopoietic-specific inactivation of *Tet2* (*Tet2*KO) bone marrow were fed WD for 6-9 weeks followed by isolation of atria and tissue digest for single cell suspension (4500U/ml collagenase I, 125U/ml collagenase XI, 60U/ml DNaseI, 60U/ml hyaluronidase). Pooled digest of atria were characterized by flow cytometry (FACS CantoII, BD Bioscience). Donor hematopoietic cells are CD45.2$^+$, recipient hematopoietic cells are CD45.1$^+$. Neutrophils are CD11b$^+$ Ly6G^{hi}, macrophages are CD11b$^+$ Ly6G^{lo} F4/80^{hi}, monocytes are CD11b$^+$ Ly6G^{hi} F4/80^{lo}. Statistical test: Two-tailed Student's t-test for all comparisons. KO indicates knockout; TET2, tet methylcystosine dioxygenase 2; WD, Western diet; and WT, wild-type.

Parameter	Conduction	WT (±SD)	*Tet2*KO (±SD)	*P*
**Surface ECG**	QRS (ms)	27 ± 2.6	28 ± 2.4	0.26
	PR (ms)	47.6 ± 6.6	47.6 ± 5.6	0.98
	BCL (ms)	128.9 ± 15.5	128 ± 10.6	0.88
**Basic Electrophysiology**	WCL (1:1) (ms)	76.1 ± 1.7	78.4 ± 6.1	0.26
	2:1 CL (ms)	56.2 ± 3.8	58.7 ± 5.8	0.09
	SNRT (80) (ms)	181.6 ± 25.4	189.5 ± 51.6	0.67
	AERP (ms)	29.78 ± 9.28	17.9 ± 5.3	0.003
	AV ERP (ms)	46.1 ± 5.6	48 ± 8.9	0.59
	V ERP (ms)	28 ± 5.8	28 ± 4	1.00
**Optical Mapping**	RA CV (m/s)	0.68 ± 0.22	0.525 ± 0.09	0.06
	LA CV (m/s)	0.68 ± 0.20	0.887 ± 0.24	0.16
	RA APD50 (ms)	12.7 ± 2.01	10.35 ± 1.66	0.015
	RA APD70 (ms)	19.5 ± 3.22	16.53 ± 2.65	0.047
	RA APD90 (ms)	23.31 ± 3.40	20 ± 2.89	0.04

Supplemental Table 3. Hematopoietic-specific loss of *Tet2* does not alter cardiac conduction. Lethally irradiated *Ldlr*$^{-/-}$ mice transplanted with WT or hematopoietic-specific inactivation of *Tet2* (*Tet2*KO*)* bone marrow were fed WD for 6-9 weeks followed by terminal EPS and optical mapping. Mean ± standard deviation (SD) shown. Statistical test: Two-tailed Student's t-test for all comparisons. AERP indicates atrial effective refractory period; APD, action potential duration. AV, atrio-ventricular; BCL, basic cycle length; CV, conduction velocity; CL, cycle length; EPS, electrophysiology studies; KO, knockout; LA, left atrial; RA, right atrial; SNRT, sinus node recovery time; TET2, tet methylcystosine dioxygenase 2; WD, WCL, Wenckebach cycle length; Western diet; and WT, wild-type.
