# Supplementary material for: Clonal Hematopoiesis of Indeterminate Potential With Loss of Tet2 Enhances Risk for Atrial Fibrillation Through Nlrp3 Inflammasome Activation
Source: Circulation. 2024 Feb 15;149(18):1419–34. doi: 10.1161/CIRCULATIONAHA.123.065597 (PMC11058018; doi:10.1161/CIRCULATIONAHA.123.065597)

Clonal hematopoiesis of indeterminate potential with loss of *Tet2* enhances risk for atrial fibrillation through macrophage mediated *Nlrp3* inflammasome activation

Uncropped Gel Blots

Representative figure lanes enclosed within dashed box

Full Unedited Gel for Figure 2G

Nlrp3

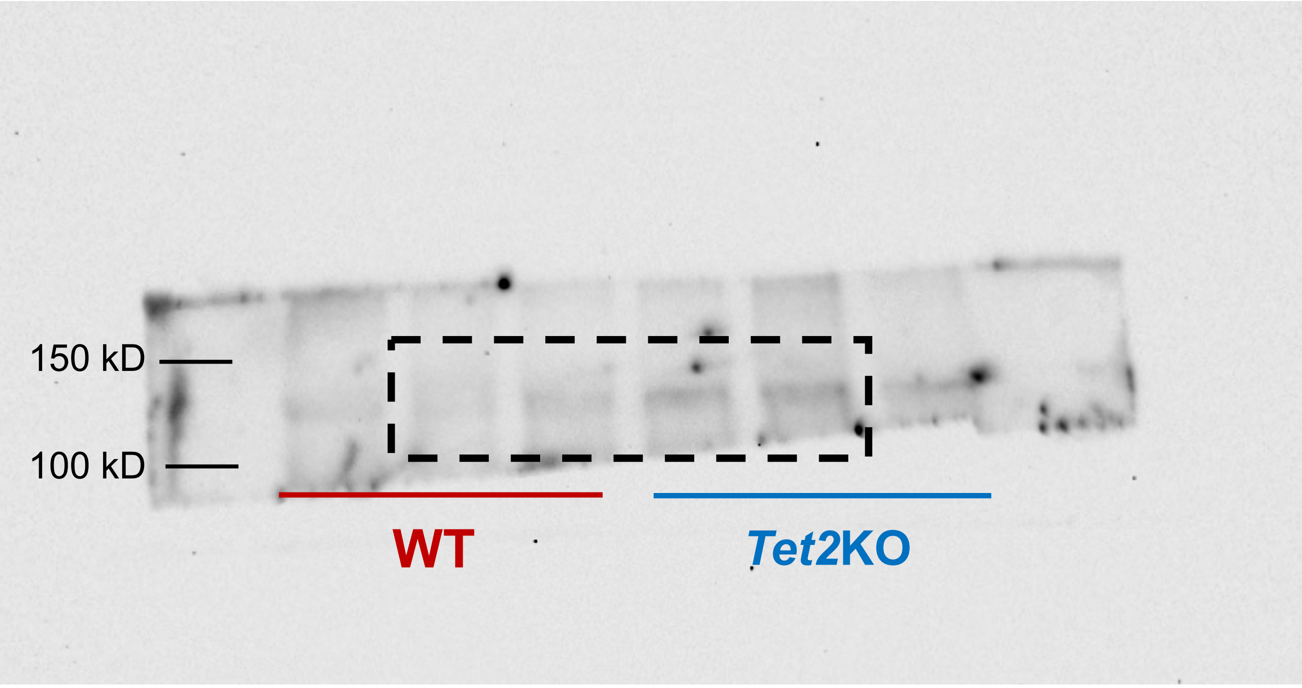

Vinculin

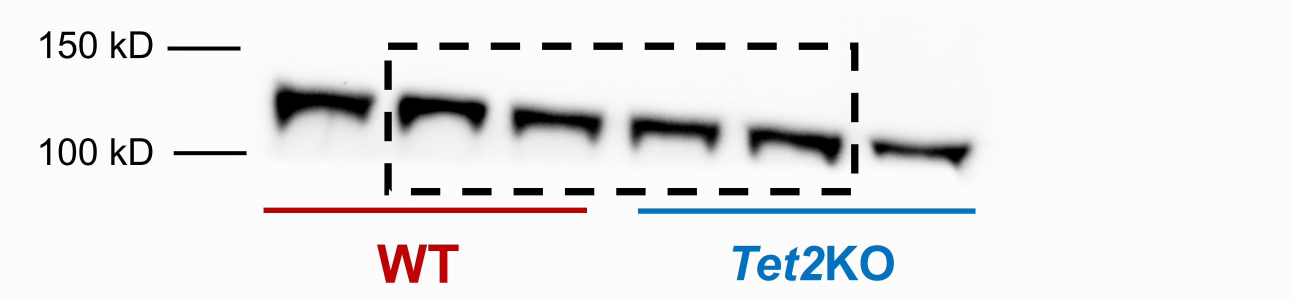

Full unedited gel for Figure 5B

pCaMKII

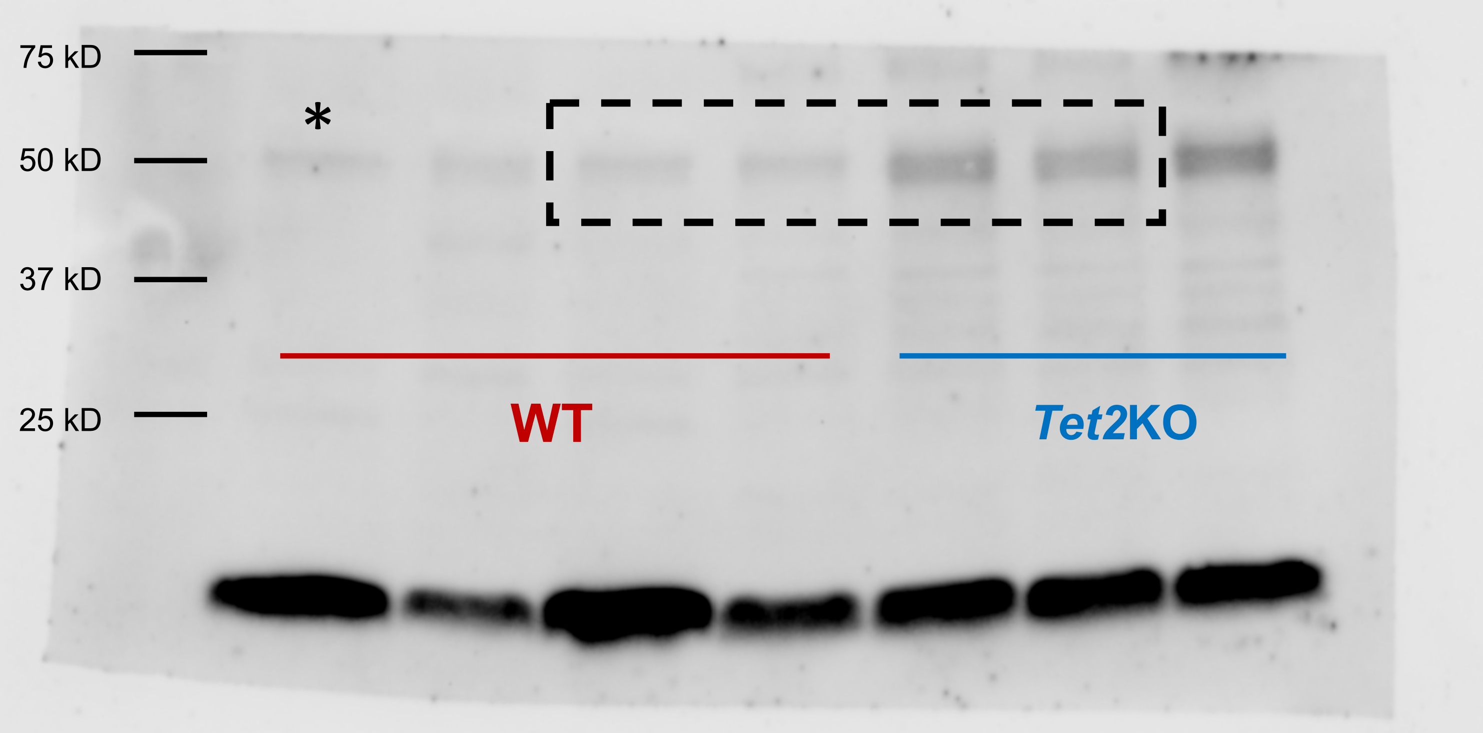

Vinculin

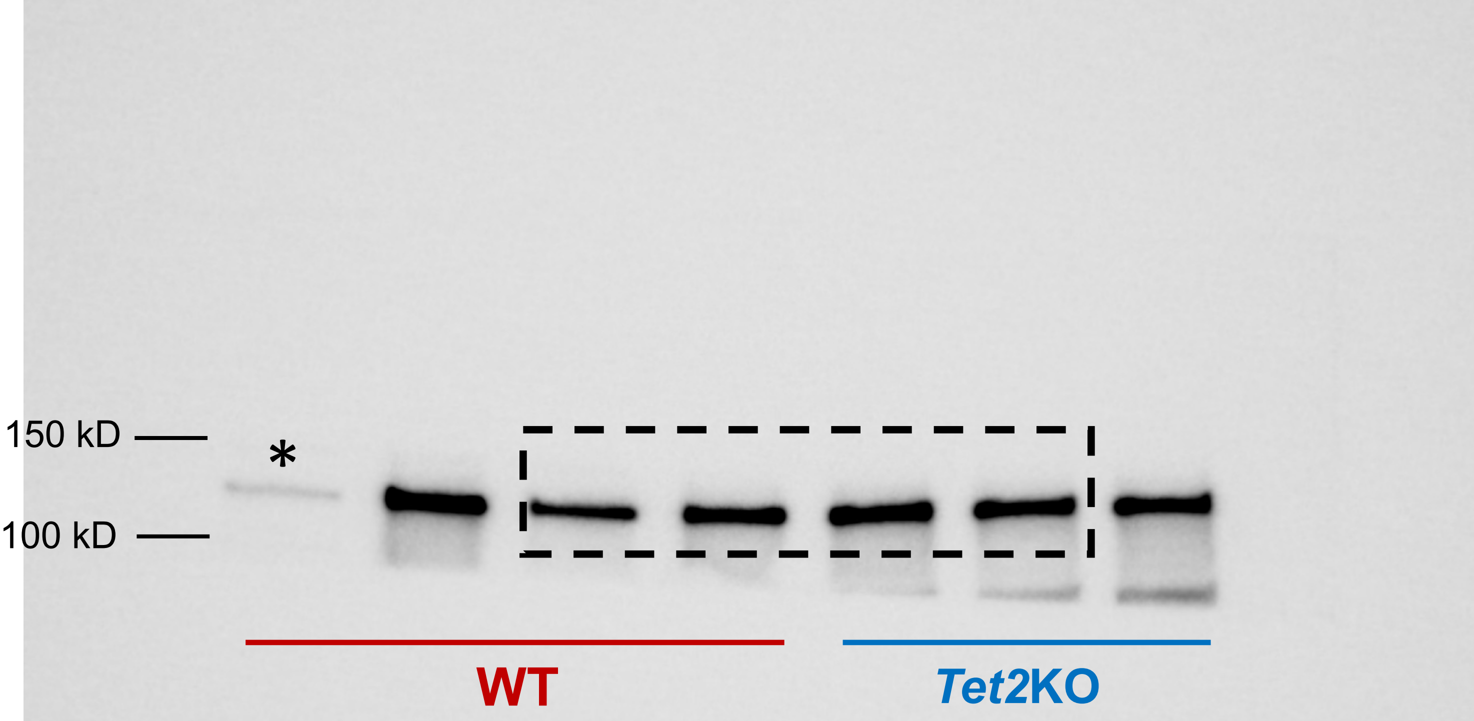

* Erroneous loading, excluded in quantitation.

Full unedited gel for Figure 5C

pCaMKII

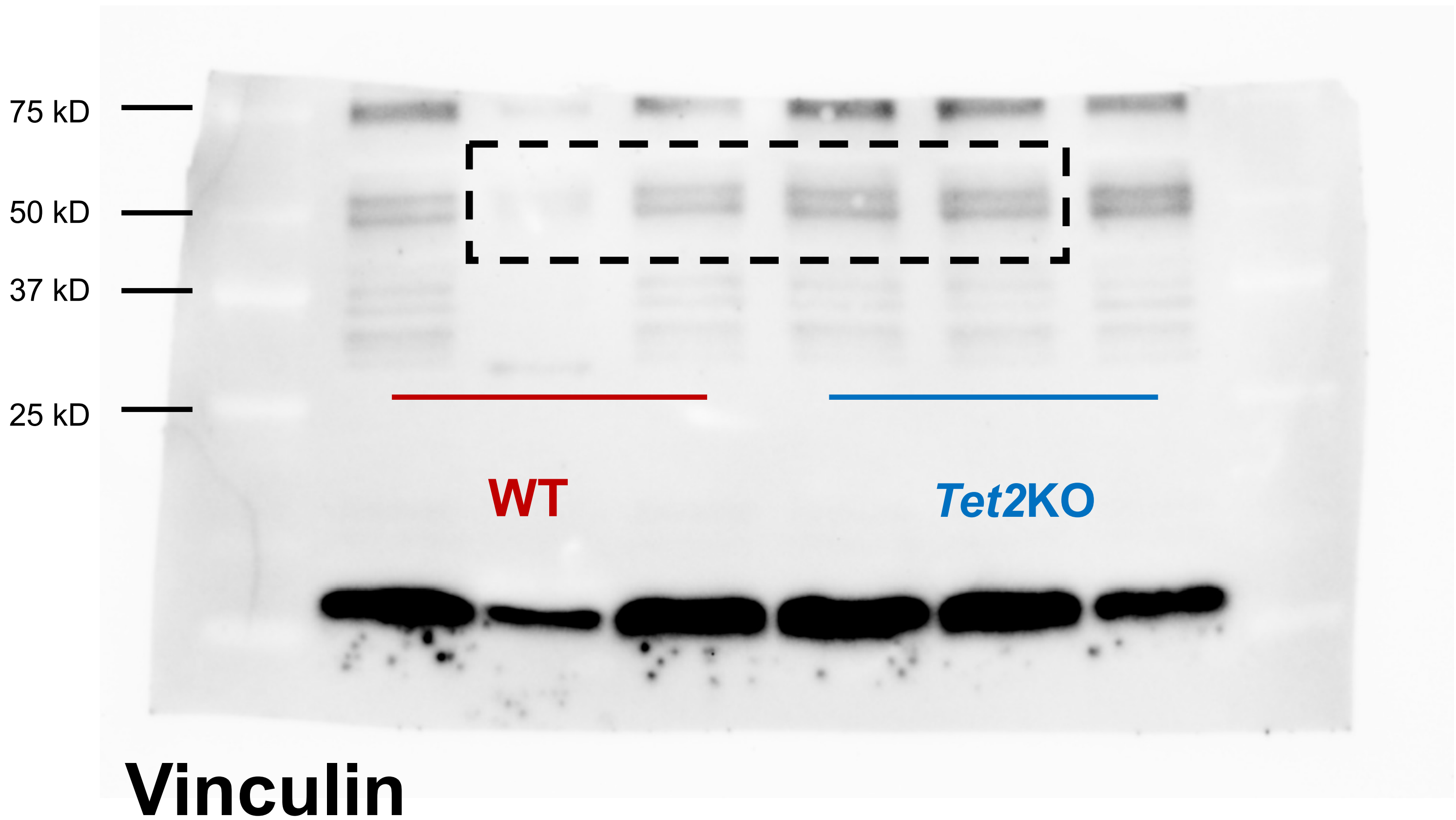

Vinculin

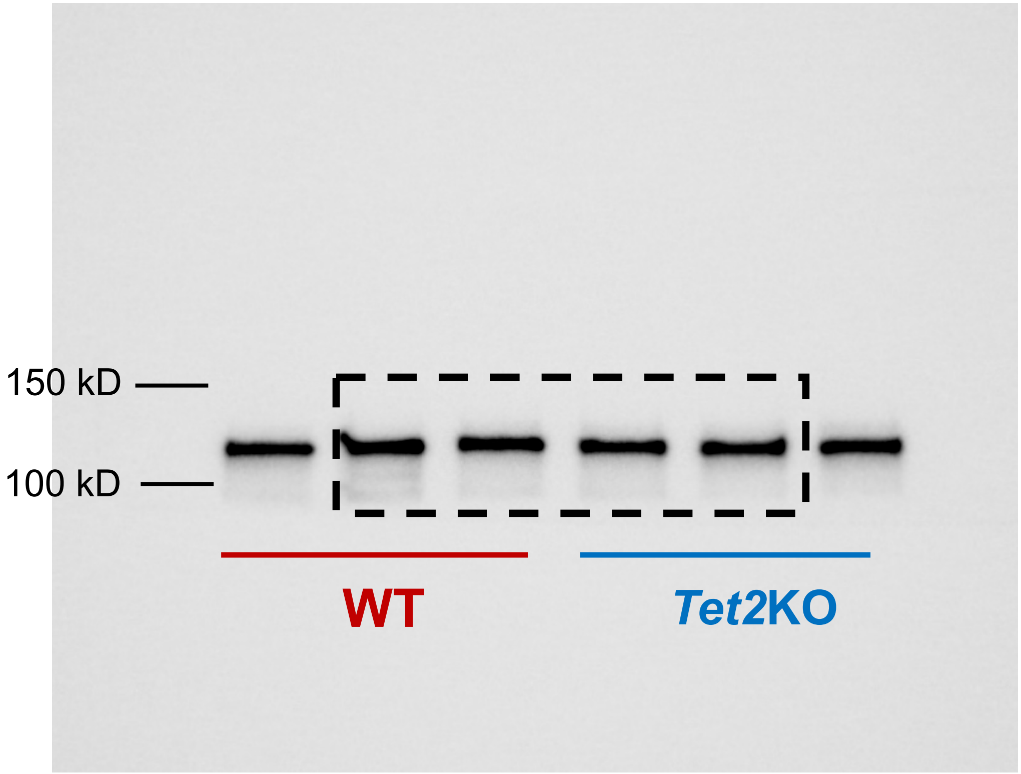

Full unedited gel for Supp. Figure 6A

Caspase 1 + p20

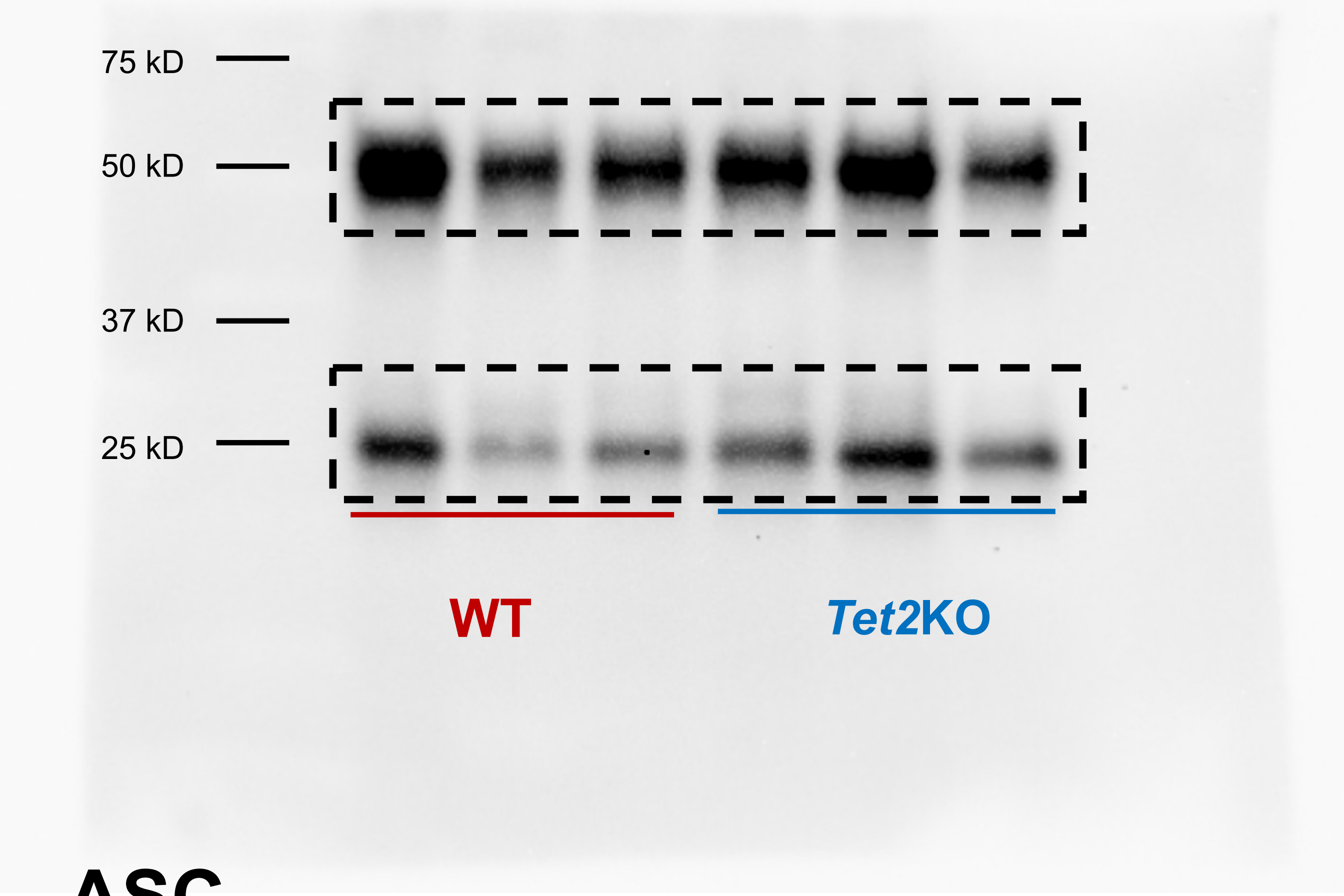

ASC

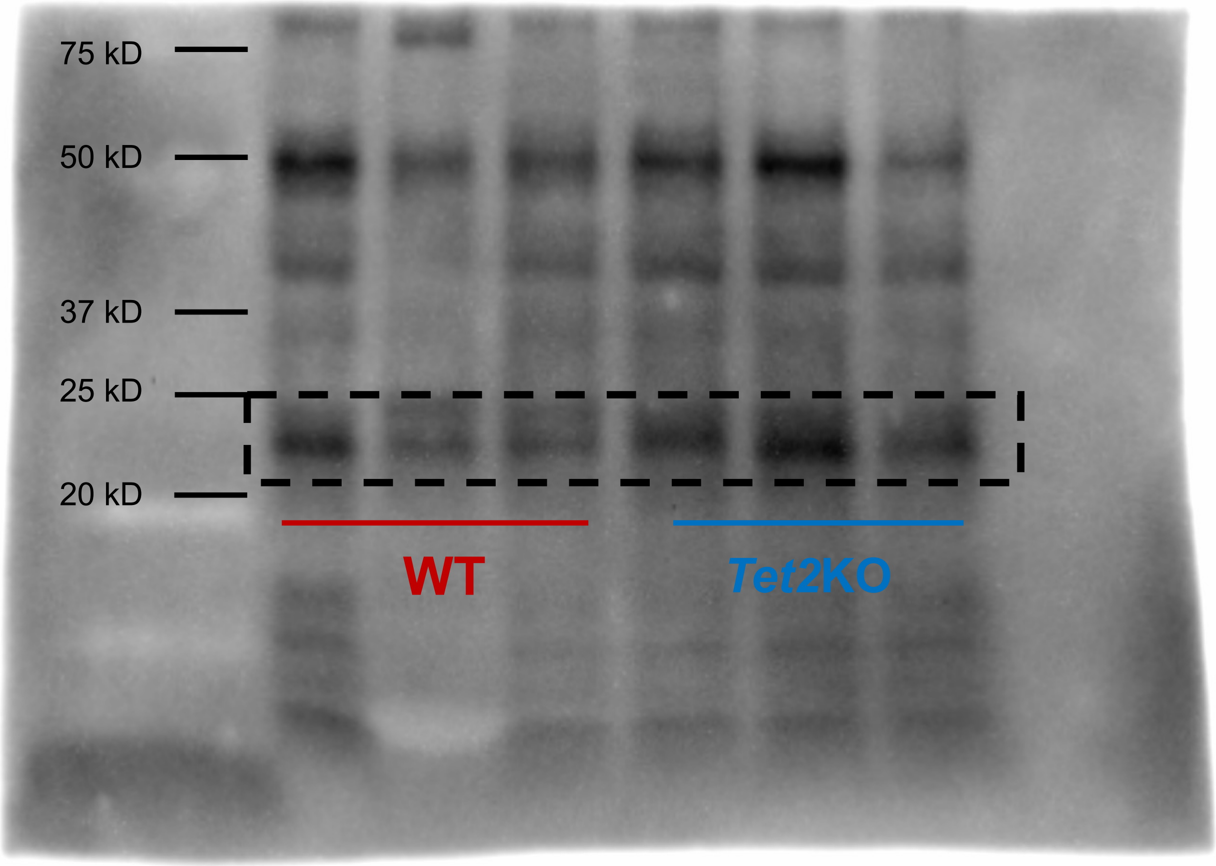

Full unedited gel for Supp. Figure 6A (cont'd)

Vinculin

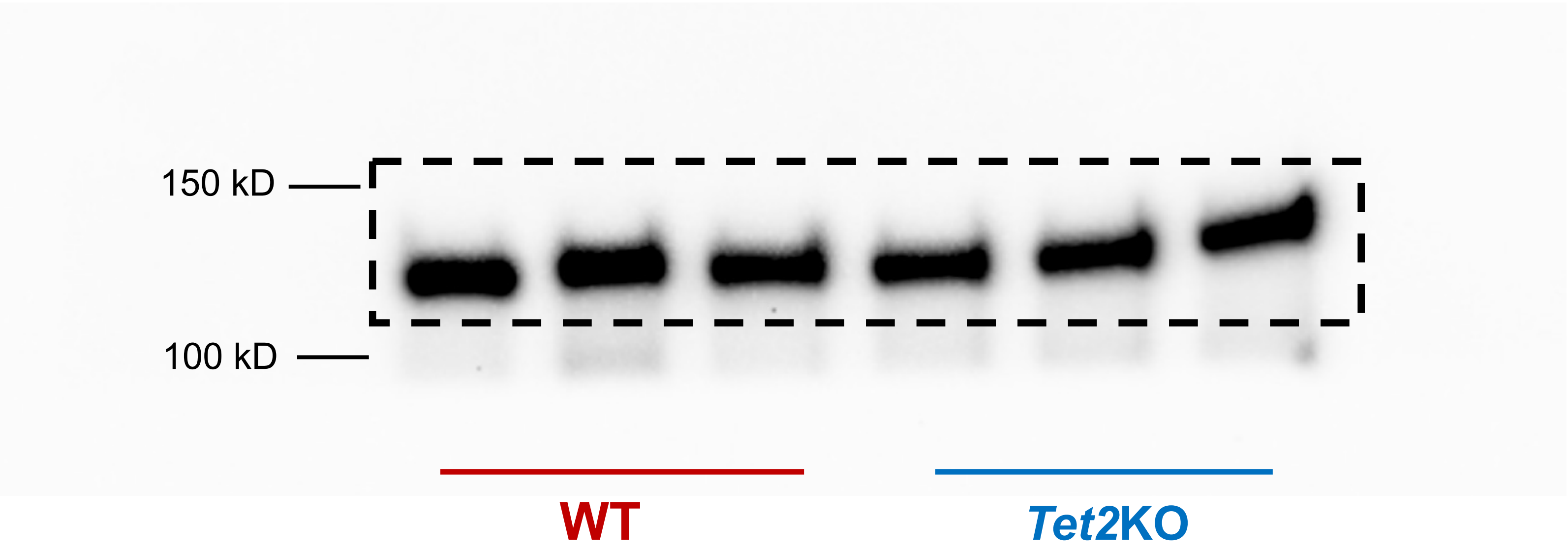

Full unedited gel for Supp. Figure 6B

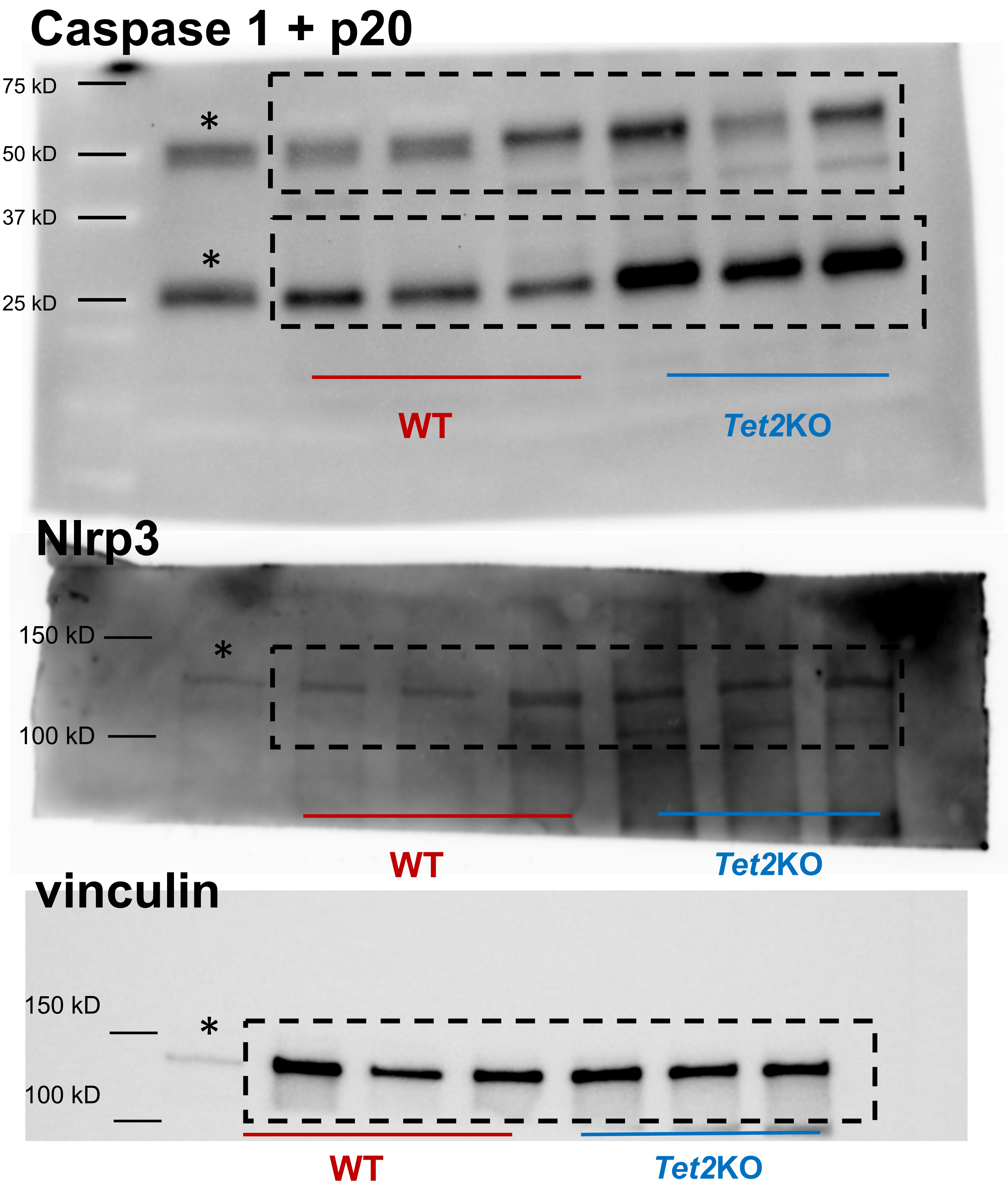

* Erroneous loading, excluded in quantitation.

Full unedited gel for Supp. Figure 6B (cont'd)

ASC

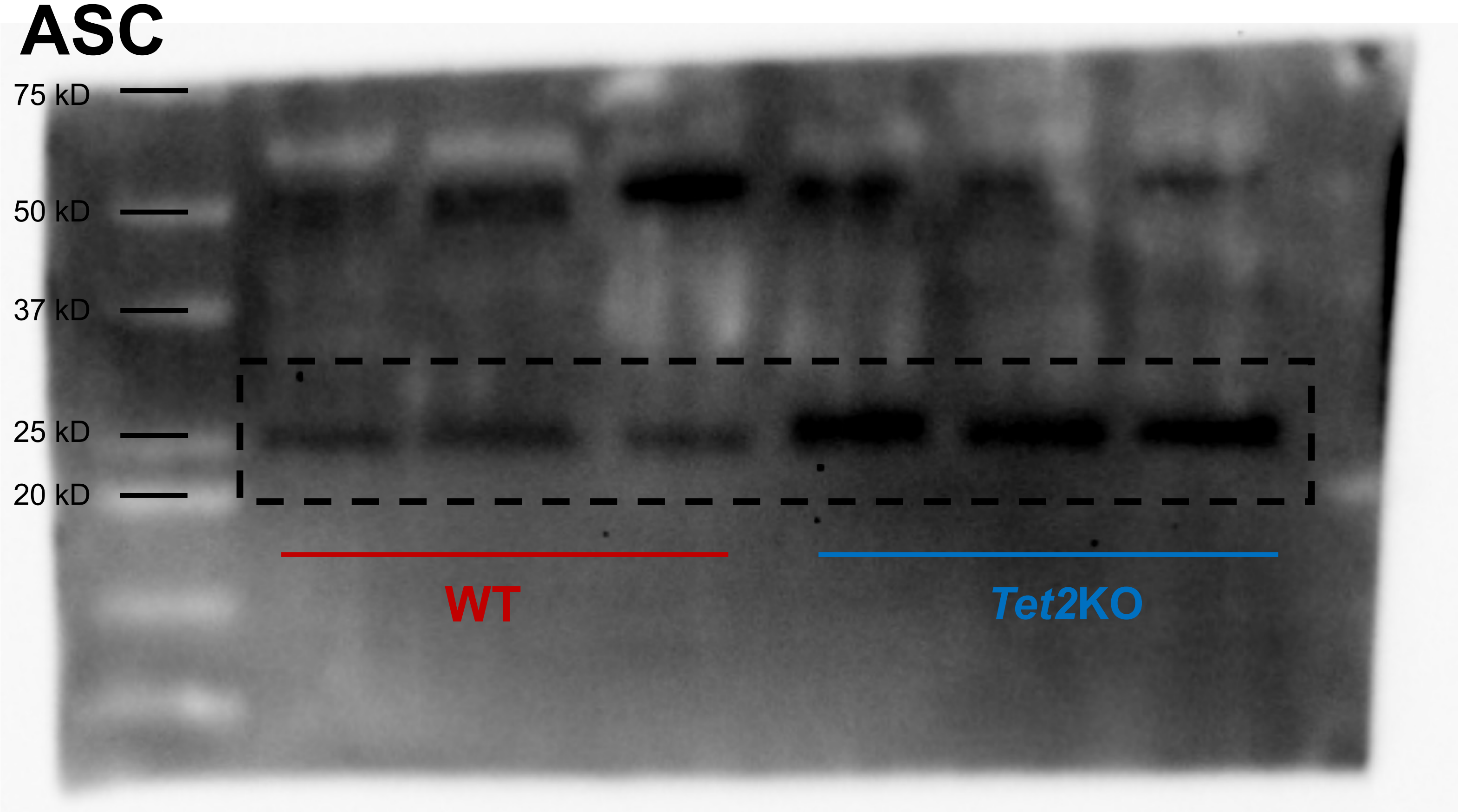

Vinculin

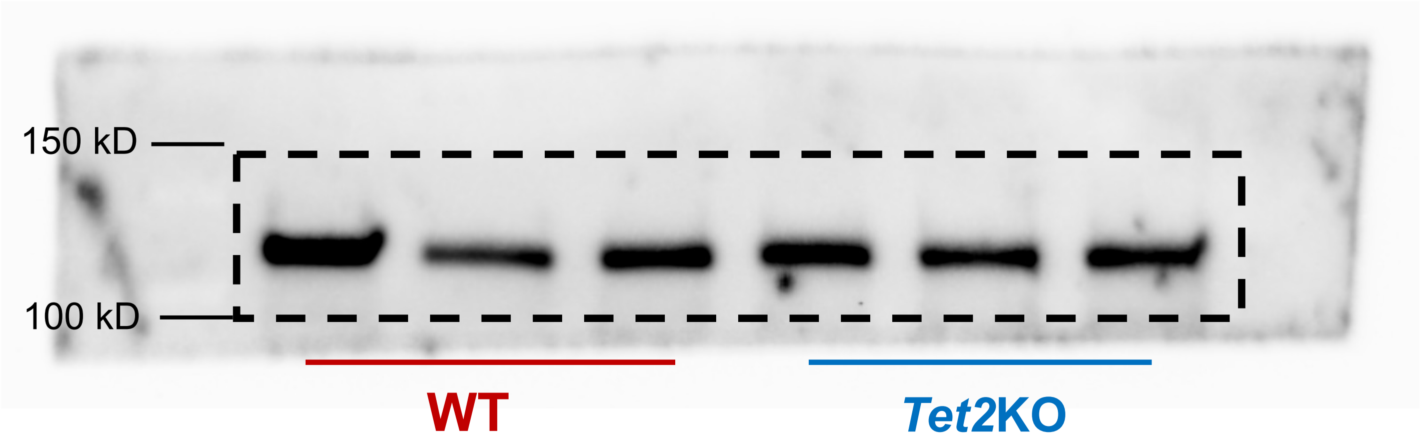

Full Unedited Gel for Supp. Figure 6C

Nlrp3

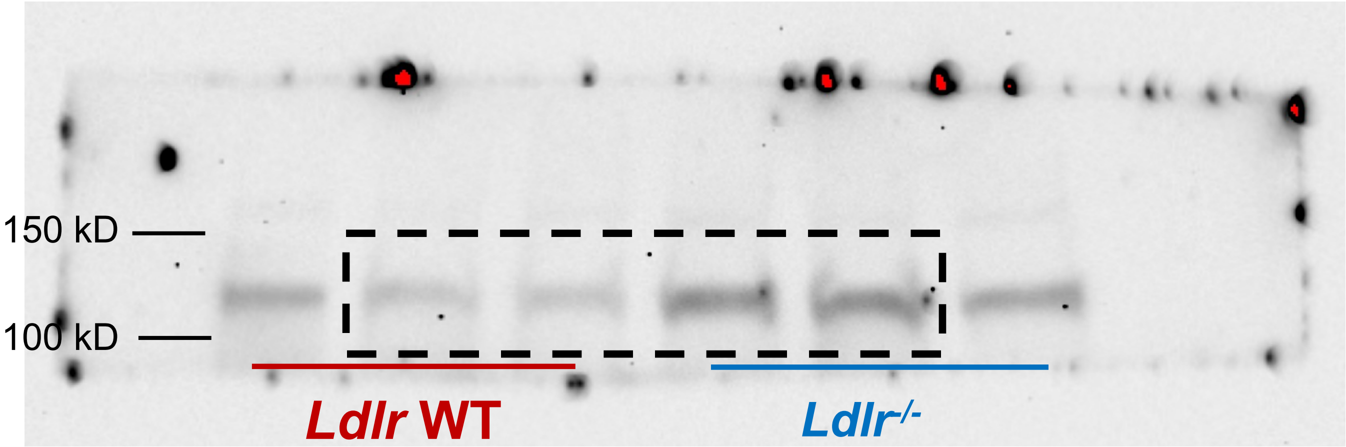

Vinculin

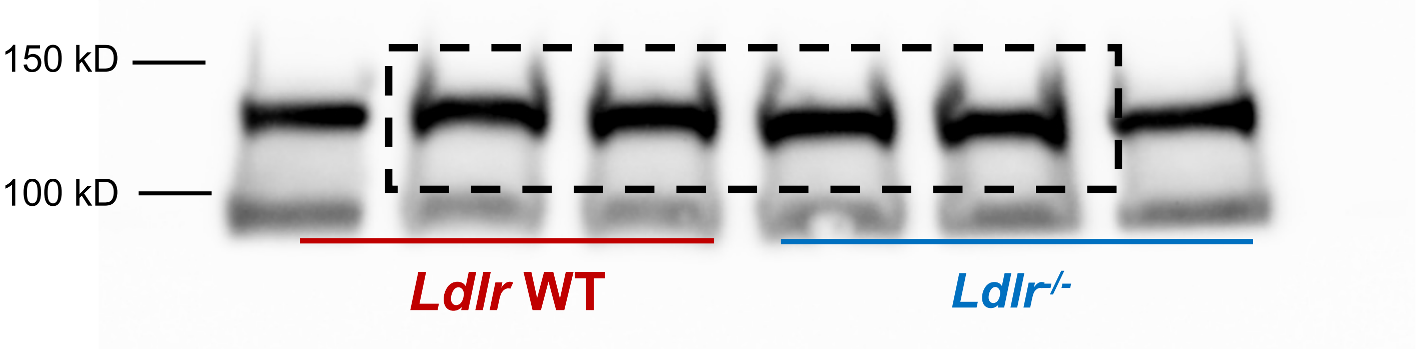

Supplement: Supplementary file 2 [file cir-149-1419-s002.pdf]
